# Regulator of G-protein signaling 1 critically supports CD8^+^ T_RM_ cell-mediated intestinal immunity

**DOI:** 10.3389/fimmu.2023.1085895

**Published:** 2023-04-20

**Authors:** Diego von Werdt, Bilgi Gungor, Juliana Barreto de Albuquerque, Thomas Gruber, Daniel Zysset, Cheong K. C. Kwong Chung, Antonia Corrêa-Ferreira, Regina Berchtold, Nicolas Page, Mirjam Schenk, John H. Kehrl, Doron Merkler, Beat A. Imhof, Jens V. Stein, Jun Abe, Gleb Turchinovich, Daniela Finke, Adrian C. Hayday, Nadia Corazza, Christoph Mueller

**Affiliations:** ^1^ Division of Experimental Pathology, Institute of Pathology, University of Bern, Bern, Switzerland; ^2^ Department of Gastrointestinal Health, Immunology, Nestlé Research, Lausanne, Switzerland; ^3^ Department of Pathology, Division of Clinical Pathology, University & University Hospitals of Geneva, Geneva, Switzerland; ^4^ National Institute of Allergy and Infectious Diseases, Bethesda, MD, United States; ^5^ Department of Pathology and Immunology, Centre Medical Universitaire, University of Geneva, Geneva, Switzerland; ^6^ Department of Oncology, Microbiology and Immunology, University of Fribourg, Fribourg, Switzerland; ^7^ Department of Biomedicine, and University Children’s Hospital Basel, University of Basel, Basel, Switzerland; ^8^ Peter Gorer Department of Immunobiology, School of Immunology and Microbial Sciences, King's College London, London, United Kingdom; ^9^ The Francis Crick Institute, London, United Kingdom

**Keywords:** regulator of G-protein signaling-1, T-cell differentiation, TRM cells, intestinal listeria monocytogenes infection, immunosurveillance

## Abstract

Members of the Regulator of G-protein signaling (Rgs) family regulate the extent and timing of G protein signaling by increasing the GTPase activity of Gα protein subunits. The Rgs family member *Rgs1* is one of the most up-regulated genes in tissue-resident memory (T_RM_) T cells when compared to their circulating T cell counterparts. Functionally, Rgs1 preferentially deactivates Gαq, and Gαi protein subunits and can therefore also attenuate chemokine receptor-mediated immune cell trafficking. The impact of *Rgs1* expression on tissue-resident T cell generation, their maintenance, and the immunosurveillance of barrier tissues, however, is only incompletely understood. Here we report that Rgs1 expression is readily induced in naïve OT-I T cells *in vivo* following intestinal infection with *Listeria monocytogenes*-OVA. In bone marrow chimeras, *Rgs1* ^-/-^ and *Rgs1* ^+/+^ T cells were generally present in comparable frequencies in distinct T cell subsets of the intestinal mucosa, mesenteric lymph nodes, and spleen. After intestinal infection with *Listeria monocytogenes*-OVA, however, OT-I *Rgs1* ^+/+^ T cells outnumbered the co-transferred OT-I *Rgs1^-^* ^/-^ T cells in the small intestinal mucosa already early after infection. The underrepresentation of the OT-I *Rgs1* ^-/-^ T cells persisted to become even more pronounced during the memory phase (d30 post-infection). Remarkably, upon intestinal reinfection, mice with intestinal OT-I *Rgs1* ^+/+^ T_RM_ cells were able to prevent the systemic dissemination of the pathogen more efficiently than those with OT-I *Rgs1* ^-/-^ T_RM_ cells. While the underlying mechanisms are not fully elucidated yet, these data thus identify *Rgs1* as a critical regulator for the generation and maintenance of tissue-resident CD8^+^ T cells as a prerequisite for efficient local immunosurveillance in barrier tissues in case of reinfections with potential pathogens.

## Introduction

Infections with viral or intracellular bacterial pathogens frequently occur at mucosal surfaces and elicit strong adaptive CD8^+^ T cell responses. During the successful pathogen eradication, most effector CD8^+^ T cells are eventually eliminated by to apoptosis induction during the contraction phase (1, 2). Nonetheless, a population of antigen-specific memory precursor effector cells (MPEC) can survive and locally differentiate into long-lived tissue-resident memory CD8^+^ T cells (T_RM_ cells). Upon local reinfection, these CD8^+^ T_RM_ cells provide superior protection compared to circulating memory CD8^+^ T cells upon local reinfection (3–6).

Locally re-activated CD8^+^ T_RM_ cells are critical for the induction of a tissue-wide state of alert, activation of local vascular endothelial cells, maturation of antigen-presenting cells, and the recruitment of circulating innate and adaptive effector cells (7–9). Accordingly, the appropriate localization of CD8^+^ T_RM_ cells at the site of primary infection ensures rapid pathogen detection and control. However, despite considerable efforts, it remains incompletely understood how CD8^+^ T_RM_ cell differentiation and retention is regulated at the site of infection.

CD8^+^ T_RM_ cells from barrier tissues differentially express members of the *Regulator of G-protein signaling* (*Rgs)* gene family when compared to their circulating counterparts (10). Using serial analysis of gene expression (SAGE) more than 20 years ago, *Rgs1* mRNA was identified as one of the most prominently expressed transcripts in intraepithelial lymphocytes (IEL) of the small intestine (SI) in mice (11). These observations suggested a potential link between T cell tissue residency and the expression of *Rgs* gene family members, notably Rgs1. The interactions between Rgs1 protein with the Gαi- (or Gαq) subunit of G-Protein-Coupled Receptors (GPCR) stabilize the [G_α_-GTP → G_α_-GDP + P_i_] transition state, thereby increasing the intrinsic hydrolysis rate of the G_α_-subunit resulting in an accelerated GTP → GDP + P_i_ conversion (12). This mechanism significantly increases GPCR desensitization and thereby regulates downstream signal transduction (12). Moreover, Rgs1-controlled GPCR signaling is critical for T cell-mediated immunity but also appears to be involved in the development of CD4 T cell-driven chronic intestinal inflammation (13). Together with previous findings, it was postulated that Rgs-regulated chemokine receptor signaling might be involved in controlling T cell migration and retention *vs*. egress from non-lymphoid tissues (10, 13).

The main aim of the present study was to determine the impact of Rgs1 on the local generation and maintenance of antigen-specific CD8 T_RM_ cells in the intestinal mucosa and to identify its relevance for an optimal local immunosurveillance to prevent systemic spreading of a pathogen following intestinal reinfection.

To directly address the impact of the *Rgs* gene family members in the differentiation of tissue-resident CD8^+^ T cells, we first compared the expression profile of selected Rgs genes in bona fide tissue-resident *vs*. circulating T cell subsets in mice under homeostatic conditions. In line with previous reports in mice and human (4, 11, 14, 15) the expression of the Rgs1 gene was found to be consistently elevated in small intestinal CD8^+^ T_RM_ cell subsets. Upon oral infection with *Listeria monocytogenes-OVA (Lm-OVA)*, *Rgs1* mRNA expression was rapidly further upregulated in small intestinal antigen-specific CD8^+^ T cells together with other critical components of the T_RM_ cell-associated genetic signature (14). Genetic deletion of *Rgs1* in antigen-specific CD8^+^ T cells significantly impaired their accumulation at the site of intestinal infection. In *Lm-OVA* infected mice that initially received equal numbers of OT-I R*gs1^+/+^* and OT-I *Rgs1^-/-^* cells, the antigen-specific, *Rgs1*-deficient CD8^+^ T_RM_ cells were underrepresented in the small intestinal mucosa from day 6 post-infection (p.i.) until the end of the observation period, i.e., on day 30 p.i. Finally, by utilizing a heterologous *in vivo* re-challenge model, we show that genetic deletion of *Rgs1* in antigen-specific CD8^+^ T_RM_ cells resulted in significantly impaired intestinal barrier immunity, accompanied by increased systemic pathogen dissemination. Collectively, these findings identify *Rgs1* as a critical regulator of local CD8^+^ T_RM_ cell-mediated immunity in the small intestine.

## Material and methods

### Mice, virus, and bacteria

Recombinant *Lm-OVA* (16) and r*LCMV-OVA* (strain V455 with mutated NP396 epitope (17) were described before. C57BL/6JRj mice were purchased from the central animal facility, University of Bern, or obtained from Janvier Labs, France. CD45.1 mice (B6.SJL-Ptrc<a> Pepc<b>/BoyJ) were originally obtained from Jackson Laboratories; USA (JAX:002014). OT-I mice (C57BL/6-Tg(Tcra/Tcrb)1100Mjb/J) were obtained from the Swiss Immune Mouse Repository (University of Zurich). The CD11c-EYFP mouse strain (18) expressing yellow fluorescent protein (YFP) under the transcriptional control of the mouse integrin alpha X (Cd11c) promoter was initially obtained from The Jackson Laboratory (JAX stock #008829), OT-I CD45.1/.2 mice were generated by crossing CD45.1 and OT-I mice (B6.SJL-Ptrc<a> Pepc<b>/BoyJ x C57BL/6J-Tg(C57BL/6-Tg(Tcra/Tcrb)1100Mjb/J). *Rgs1^-/-^* OT-I mice (B6.129P2-Rgs1<tm1Jhk>/x B6.SJL-Ptrc<a> Pepc<b>/BoyJ) were generated by back-crossing of OT-I mice with Rgs1^-/-^ mice (B6.129P2-Rgs1<tm1Jhk>) (19) For all experiments mice between 10 - 12 weeks of age were used. Mice were kept at the central animal facilities of the University of Bern, except for intravital 2-photon microscopy, which was done at the respective facility at the University of Fribourg, Switzerland. All experimental procedures were approved by the Cantonal Committees for Animal Experimentation and conducted according to federal guidelines.

### Generation of mixed bone marrow chimeras

CD45.1 x CD45.2 recipient mice were irradiated twice within 4h with a total dose of 650 cGy. For the reconstitution, bone marrow cells from wild-type CD45.1 mice and *Rgs1* ^-/-^ (CD45.2) mice were used. Bone marrow cell suspensions were counted, and wild-type *Rgs1* ^+/+^ and *Rgs1* ^-/-^ cells were mixed 1:1 at a final concentration of 3x10^7^ cells/ml in sterile PBS. From this suspension, 400μl/mouse (total: 1.2x10^7^ cells) were injected i.v. into previously irradiated recipients. The mice were treated with antibiotics in drinking water for 2 weeks (Bactrim, Roche, Switzerland, oral suspension, diluted 1:200); 2.5ml/250ml Baytril 2,5%, Bayer, Germany). For mixed bone marrow chimera experiments, the exact ratio of transferred CD45.1 *Rgs1* ^+/+^ and CD45.2 *Rgs*1^-/-^ bone marrow cells was determined by flow cytometry. Frequencies of wild type and *Rgs*1^-/-^ T lymphocyte cells were then normalized to the inoculum.

### Adoptive transfers and infections

Immune chimeras were generated by (co)-transferring 2.5x10^5^ OT-I *Rgs1^+/+^* (CD45.1 x CD45.2) and/or OT-I cells *Rgs1^-/-^* (CD45.2) into naïve CD45.1 recipient mice. For *Lm-OVA* infections, the mice were i.g. inoculated with 1x10^9^ CFU one day before cell transfer. For *rLCMV-OVA* infections, mice were i.p. inoculated with 1x10^5^ PFU one day after cell transfer. We deliberately administered *Lm*-OVA by an intragastric gavage (i.g), rather than by oral feeding: infection with Lm-OVA *via* the i.g. route, favors the induction of CCR9 expressing gut homing effector CD8αβ+ T cells whereas the oral administration of Lm-OVA favors the systemic dissemination of antigen-specific T cells after their priming in the draining submandibular lymph nodes as recently demonstrated by us (20).

### *In vivo* CD8^+^ T cell depletion and FTY720 treatment

CD8^+^ T cells were depleted from the circulation by i.p. injection of anti-CD8α monoclonal antibody (mAb) (450μg anti-CD8α/mouse, clone YTS 169.4, BioXCell, West Lebanon, USA, dissolved in 200μl PBS). Mice were injected i.p. with FTY720 (25μg/mouse, dissolved in 100μl 0.9% NaCl) Sigma-Aldrich, St. Louis, USA) on day -2, day 0, and day 2 after oral re-challenge.

### Determination of Lm-OVA titers from extra-intestinal organs

*Lm*-OVA titers were assessed in mLN, spleen and liver. The organs were removed from a euthanized animal under sterile conditions and transferred into 2ml tubes containing a steel bead and 1ml PBS + 0.1% Tween20 (Sigma-Aldrich, St. Louis, USA). The organs were weighed and lysed using a QiaTissueLyzer (Qiagen, Venlo, Netherlands) at 25Hz, 3min. From the lysates 1:5, 1:50, and 1:500 dilutions were prepared. The dilutions were plated in individual wells of BHI agar-containing 6-well plates. The plates were incubated for 2 days at 37°C before the colonies were counted. *Lm*-OVA titers were calculated per gram of tissue.

### *In vivo* intravascular T cell labeling

I.v. labeling was performed as previously published (21). For the discrimination of circulating and tissue-resident T cells, mice were injected i.v. with a fluorochrome-conjugated anti-CD45 antibody (2μg/mouse, anti-CD45-BV711 or -Pe-Cy5, clone 30-F11, Biolegend, San Diego, USA). Mice were euthanized 3 min after the injection.

### Lymphocyte isolation

Lymphocytes from the spleen and mesenteric lymph nodes (mLN) were isolated as follows. Spleen and mLN were dissected, and the organs were smashed through a 40μm cell strainer. For the isolation of splenocytes, erythrocytes were lysed in an ACK lysis buffer. The cells were resuspended and stored on ice for further processing.

Small intestinal IEL and LPL were isolated as previously described with minor modifications (22–24). The small intestine was opened longitudinally, and feces were removed by swirling the tissue in Hanks í balanced salt solution (HBSS) + 2% horse serum (HS). IEL were isolated by incubating the small intestinal tissue for 15min in Stripping Buffer (1×HBSS, 2mM dithiothreitol (DTT), 0.5mM EDTA, HBSS + 2% HS, Sigma- Aldrich, St. Louis, USA). IEL were enriched using a Percoll gradient (40% and 70%, GE Healthcare Bio-science, Uppsala, Sweden). IEL were collected from the interphase and kept on ice for further processing. The remaining tissue pieces were further processed for the isolation of lamina propria lymphocytes (LPL). Tissue pieces were transferred into Digestion Buffer (HBSS + 2% HS, 50-100mg/100ml Collagenase IV (Sigma-Aldrich, St. Louis, USA), 1.3 mM CaCl2, 0.5 mM MgCl2, 0.6 mM MgSO4, Dnase1 (20μg/100ml, Roche Diagnostics, Rotkreuz, Switzerland) and incubated for 20min. The LPL single-cell suspension was pelleted, resuspended in HBSS + 2% HS and stored on ice for further processing.

### Flow cytometry and cell sorting

Before staining, all cells were treated with Fc-block (anti-CD16/CD32, clone 93, 1μg/ml, Biolegend, San Diego, USA) in PBS, for 10min, on ice. Cells were washed and stained in HBSS + 2% HS on ice for 30min. After staining the cells were washed and fixed using the BD Cell Fix kit (BD Biosciences, USA). For all analyses, dead cells were excluded using viability dyes (LIVE/DEAD fixable blue dead stain kit, Thermo Fisher Scientific, Carlsbad, USA, Zombie Aqua fixable viability kit, Biolegend, San Diego, USA). Fluorescence-activated cell sorting (FACS sorting) was performed on a BD ARIA3 (BD Biosciences, USA) or a Moflo Astrios (BD Biosciences, USA) instrument. For flow cytometry analysis, the samples were acquired on a BD Sorp LSR2 (BD Biosciences, USA).

### *In vivo* cell proliferation

Cell proliferation was evaluated *via* 5-ethynyl-2′-deoxyuridine (EdU) incorporation in *Lm-OVA* infected mice on day 7 and day 8 p.i. For this, 1mg EdU (Merck, Darmstadt, Germany) was injected i.p. into the mice 12 and 24 h before sacrifice (2mg in total) (25–28). Mice were treated i.p. with 50 μg anti-ARTC2 nanobodies (Biolegend, San Diego, USA) 20 min before tissue collection to block the eATP/NAD^+^-induced cell death during the isolation of small intestinal lymphocytes (29). Mice were euthanized 3 min after the i.v. injection of 2μg fluorochrome-conjugated anti-CD45 antibody, and single cells were separated as previously described. EdU-incorporated S-phase cells were detected using Click-iT™ EdU Pacific Blue™ Flow Cytometry Assay Kit (Thermo Fischer Scientific, Carlsbad, USA) according to the manufacturer’s instructions. EdU-positive cells were analyzed by a BD Sorp LSR2 (BD Biosciences, USA).

### Intravital 2-photon microscopy

CD11c EYFP mice (18) or C57BL/6JRj mice received 2.5x10^5^ OT-I *Rgs1^+/+^* -tdT + 2.5x10^5^ OT-I *Rgs1^-/-^* -GFP (16) and were orally infected with 2x10^9^ *Lm-OVA* the day after as described (20). At day 8 and at day 30 p.i., the gut lumen was surgically exposed (kept in saline at 37°C), and 2-photon microscopy (2-PM) was performed with an Olympus BX50WI microscope and a TrimScope 2-PM system controlled by ImSpector software (LaVisionBiotec, Bielefeld, Germany). Before recording, Hoechst dye was injected i.v. to label nuclei. YFP or Hoechst signals were used as a reference channel for real-time offset correction to minimize tissue shift (30). Sequences of image stacks were transformed into volume-rendered four-dimensional videos using Imaris software (Bitplane, Zurich, Switzerland), which was also used for semi-automated tracking of cell motility in three dimensions. Cell centroid data were used to calculate key parameters of cell motility. Speed was defined as total track length divided by total track duration in µm/min. Instantaneous speed was defined as the speed at each time point. The arrest coefficient was derived from the percentage of time a cell is migrating below a motility threshold speed of 5 µm/min using Matlab script (R2019b, MathWorks, Natick). The meandering index was calculated by dividing displacement divided by track length.

### OT-I T cell culture

OT-I *Rgs1^+/+^* T cells were FACS sorted, and 1x10^5^ cells/well were distributed in a 96-U-bottom-well plate in 200μl cell culture media (RMPI 1640 + 2mM L-Ala/L-Glu, 1mM Sodium Pyruvate, 10mM HEPES, 1x MEM non-essential amino acids, 0.5mM 2-β-Mercaptothion, 10% FCS, 40 U/ml Penicillin, 40μg/ml Streptomycin (all Sigma-Aldrich, St. Louis, USA)) and stimulated with distinct cytokines and TCR-crosslinking mAbs. For TCR stimulation, plates were previously coated with anti-CD3ε and anti-CD28 mAb´s (anti-CD3ε: clone 145-2C11, anti-CD28: clone 37.51, both Biolegend, San Diego, USA) for 12h. The cells were treated with recombinant murine IL-2 (50 ng/ml, R&D Systems, Minneapolis, USA), IL-15 (50 ng/ml, Peprotech, London, United Kingdom), IL-33 (100 ng/ml, Peprotech, London, United Kingdom) and/or recombinant human TGFβ1 (50 ng/ml Peprotech, London, United Kingdom). The cells were incubated for 72h, at 37°C in 5% CO_2_.

### Transwell migration assay

Naïve OT-I *Rgs1^+/+^* and CD45-congenic OT-I *Rgs1* ^-/-^ CD8^+^ T cells were isolated from the spleen of the respective donor mice using a naïve CD8^+^ T cell isolation kit (Stemcell Technologies, Vancouver, Canada) according to the manufacturer’s protocol. Cells were stimulated and incubated for 72h similarly as described above. For the transwell migration assay, the cells were washed (380g, 3min, RT), and the two populations were mixed at a 1:1 ratio at a concentration of 2x10^6^ cells per ml. 100μl of this cell suspension was distributed to the upper chambers of HTS 96-well 5μm Transwells (Corning, Corning, USA). The lower/receiving chamber contained 200μl medium with different concentrations of recombinant murine CCL21 (Peprotech, London, United Kingdom) (i.e., media control, 25nM, 100nM, 200nM). The transwells were placed on the receiving chambers and incubated for 2h at 37°C, 5% CO_2_. Migrated cells were stained, mixed with AccuCheck counting beads (Thermo Fischer Scientific, Biolegend, San Diego, USA), and enumerated by CytoFlex flow cytometer (Beckman Coulter, Pasadena, USA) in the plate loader mode.

### Gene expression analysis

For multiplex gene expression analysis, a customized NanoString nCounter Custom CodeSet (NanoString Technologies, Seattle, USA) was used. For cell sample sizes larger than 1x10^5^ cells, RNA was extracted using the TRI reagent (Sigma-Aldrich, St. Louis, USA). For gene expression analysis, a minimum of 25 ng of total RNA per sample was used. For sample sizes smaller than 1x10^5^ cells, FACS sorted cells were lysed in 8μl, 33% RLT lysis buffer, containing 1% 2-β-mercaptoethanol (Qiagen, Venlo, Netherlands). 5μl per sample were used for the NanoString^®^ analysis. Only samples with a minimum of 1.2x10^4^ cells were included for analysis.

### R Studio analysis

Heatmaps, correlation plots, and principal component analysis plots were created in R Studio (Rstudio Inc., Boston, USA). Gene expression data were generated with Nanostring arrays or retrieved from the indicated, pre-existing, open-access RNA sequencing data sets (31, 32).

### Data analysis and statistics

Flow cytometry raw data were analyzed in FlowJo (BD Biosciences, USA). If required, the data were exported to Microsoft Excel (Microsoft, Redmond, USA) for further processing (e.g., normalization). NanoString raw data were imported into nSolver (NanoString Technologies, Seattle, USA). Sample quality control and normalization were performed in nSolver. Data were plotted and tested for statistical significance in GraphPad Prism (GraphPad Software, San Diego, USA).

## Results

### Resident and circulating T cell subsets display distinct expression profiles of members of the Rgs gene family

**Figure 1:**
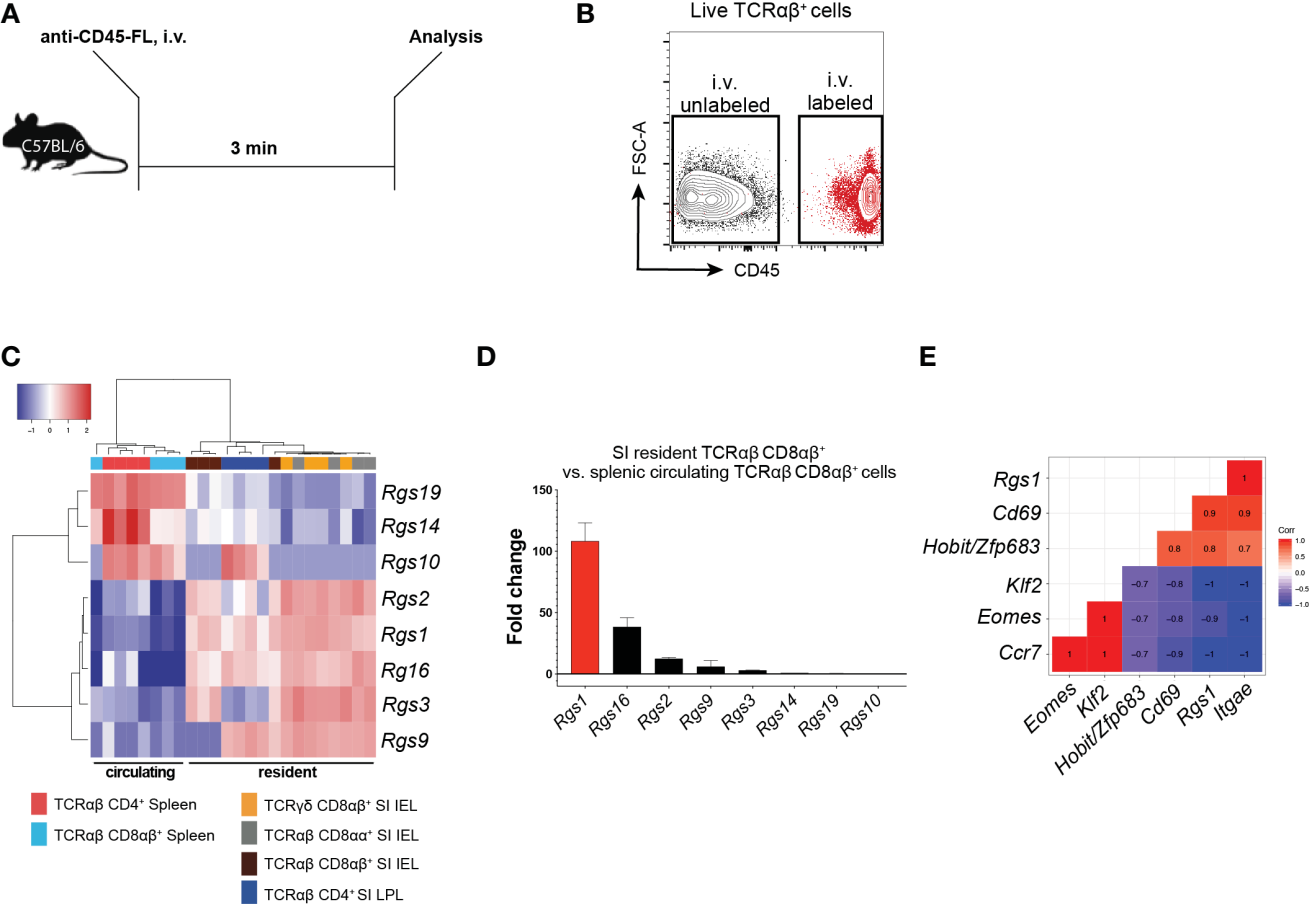
Expression of *Rgs* gene family members in small intestinal resident T cell subsets versus circulating splenic cell subsets **(A)** Experimental set-up. C57BL/6JRj mice were injected i.v. with a fluorophore (FL)-conjugated anti-CD45 mAb 3 min before euthanasia. **(B)** Representative FACS plot showing the isolation strategy for i.v. labeled, circulating *vs*. i.v. unlabeled, resident T cells. **(C)** Heat map of the *Rgs* gene expression profile of circulating and resident T cell subsets (all data points are from FACS-sorted cells pooled from 1 to 5 animals. Analyses were repeated at least 3 times with comparable results). **(D)** Fold change in the expression of *Rgs* gene members in small intestinal TCRαβ CD8αβ^+^ IEL *vs*. splenic TCRαβ CD8αβ^+^ T cells (all data points represent cells pooled from 1 to 5 animals). Analyses were repeated at least 3 times with comparable results, mean ± SEM. **(E)** Pearson correlation of *Rgs1* expression and signature genes predominantly expressed by the resident (i.e. *Itgae*, *Cd69*, *Zfp683/Hobit*) *vs*. circulating (i.e. *Klf2*, *Eomes*, *Ccr7*) memory TCRαβCD8αβ^+^ T cells (n=8, all data points represent cells pooled from 1 to 5 animals. Analyses were repeated at least 3 times with comparable results).

Several members of the *Rgs* gene family critically regulate the chemotactic recruitment of cell subsets (33). Hence, we first determined the *Rgs* gene family expression profiles of the *Rgs* gene family members in circulating and tissue-resident CD4^+^ and CD8^+^ T cell subsets (including T_RM_ cells). By injecting mice with a fluorophore-conjugated anti-CD45 monoclonal antibody (mAb) 3 min before euthanizing, we discriminated resident from circulating T cells (34) (**Figures 1A, B**). Expression of specific *Rgs gene* family members was determined in FACS-sorted circulating splenic (CD45-labeled, TCRαβ CD8αβ^+^ and TCRαβ CD4^+^) and small intestinal resident (CD45-unlabeled, TCRγδ, TCRαβ CD8αα^+^, TCRαβ CD8αβ^+^, TCRαβ CD4^+^) T cell subsets by NanoString^®^ analysis. *Rgs1*, *Rgs2*, *Rgs3*, *Rgs9*, and *Rgs16* were upregulated in the resident subsets, whereas the genes encoding *Rgs10*, *Rgs14*, and *Rgs19* were upregulated in circulating T cells (**Figure 1C**). *Rgs1* was consistently the most disproportionately expressed *Rgs* gene family member (approximately 100-fold up-regulated) in resident, compared to circulating, conventional TCRαβ CD8αβ^+^ T cells (**Figure 1D**).

CD8^+^ T_RM_ cells differentially express distinct tissue-resident signature genes compared to their circulating counterparts (including *Zfp683/Hobit, Itgae, Cd69, Eomes, Klf2, and Ccr7)* (14). Accordingly, our analyses revealed that in polyclonal small intestinal CD8αβ^+^ TCRαβ IEL, *Rgs1* expression strongly correlates with the expression of *Itgae* (r=1.0), *Cd69* (r=0.9) and *Zfp683/Hobit* (r=0.8). Furthermore, the expression of *Ccr7* (r=-1.0), *Klf2* (r=-1.0), and *Eomes* (r=-0.9) negatively correlate with *Rgs1* expression (**Figure 1E**). We subsequently validated these results by *in silico* analysis of publicly available datasets containing RNA-sequencing data of CD8^+^ T_RM_ cells generated for human CD8+ T_RM_ cells (15) and found that this *Rgs1* expression profile seen in mice also applies to human CD8^+^ T_RM_ cells. Accordingly, human CD8^+^ T_RM_ cells display similarly elevated *RGS1* mRNA expression levels compared to their circulating counterparts (**Supplementary Figures 1A, B**).

These findings thus confirm the distinct expression of Rgs gene family members in resident and circulating CD8^+^ T cells. The *Rgs1* gene was consistently found to be the most up-regulated *Rgs* gene family member in tissue-resident CD8^+^ T cells from the small intestine. Furthermore, the strong positive correlation between the expression of *Rgs1* and canonical CD8^+^ T_RM_ genes suggests that *Rgs1* may play a critical role in the acquisition and maintenance of the tissue-resident phenotype of CD8^+^ T_RM_ cells.

### Rgs1-deficiency does not impair the capacity to reconstitute the small intestinal T cell compartment

**Figure 2:**
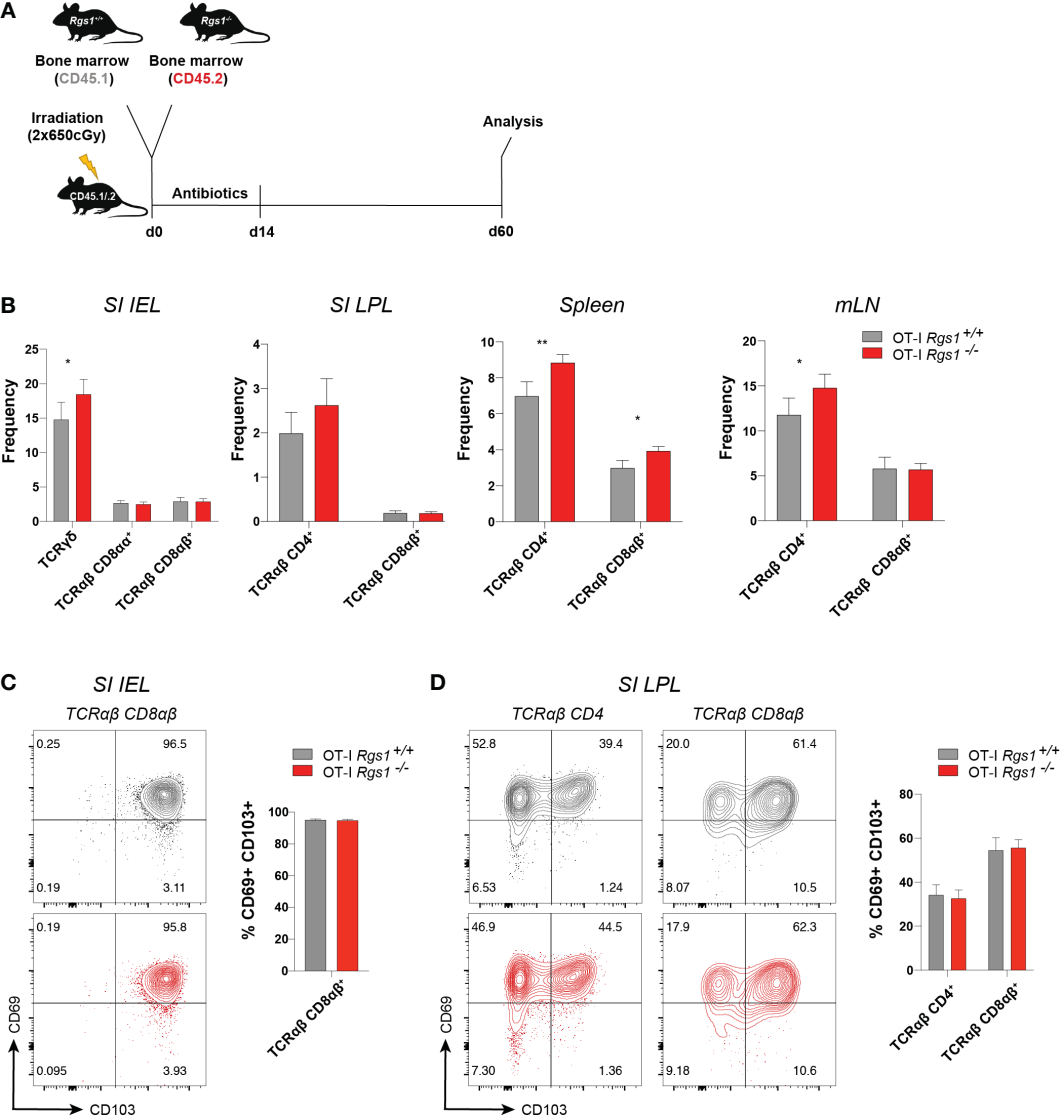
Absence of *Rgs1* does not affect the frequency of small intestinal resident and circulating T cell subsets under homeostatic conditions. **(A)** Experimental set-up. Irradiated CD45.1/.2 recipient B6 mice were reconstituted with a 1:1 mixture of congenic *Rgs1^+/+^* (CD45.1): *Rgs1^-/-^* (CD45.2) bone marrow cells. At day 60 post-reconstitution T cell subsets from different organs were isolated for analysis. **(B–D)** Frequencies of the respective T cell subsets within the isolated viable cell population in the **(B)** Small intestinal epithelium (SI IEL), lamina propria (SI LPL), spleen, and mesenteric lymph node (mLN) (n=16-21, obtained from 3 independent experiments, mean ± SEM, Wilcoxon test, *, *p <*0.05; **, *p* < 0.01; ***, *p* < 0.001). **(C, D)** Cell surface expression of T_RM_ marker CD69 and CD103 on TCRαβ T cell subsets isolated from **(C)** SI epithelium (SI IEL) TCRαβ CD4^-^ CD8αβ^+^ T cells, (TCRαβ CD8αβ) and **(D)** SI TCRαβ CD4^+^ CD8αβ^-^ T cells (TCRαβ CD4), and TCRαβ CD4^-^ CD8αβ^+^ (TCRαβ CD8αβ) LPL) (grey, OT-I *Rgs1* ^+/+^ cells; red, OT-I *Rgs1* ^-/-^ cells; n=11, pooled from 2 independent experiments, mean ± SEM, Wilcoxon test, *, *p* < 0.05; **, *p* < 0.01).

The observation that *Rgs1* was consistently and prominently expressed in tissue-resident T cells prompted us to investigate whether deficiency of *Rgs1* might affect their capacity to reconstitute the intestinal T cell compartments under homeostatic conditions. We first compared the T cell compartments in the intestinal mucosa, mLN, and spleen in *Rgs1* ^-/-^ and *Rgs1* ^+/+^ mice. As shown in **Supplementary Figure 2**, no major changes in the composition of the T cell compartments are seen in Rgs1 deficient mice. Notably, also the CD8αα^+^ TCRαβ IEL, which - at least in adult mice - mainly represent agonist-selected, self-reactive T cells (35) and are prototypic tissue-resident T cells under homeostatic conditions (36), are equally present in *Rgs1* ^-/-^ and *Rgs1* ^+/+^ mice (**Supplementary Figure 2**). To further prove that also in a competitive setting, i.e., in the presence of *Rgs1* ^+/+^ hematopoietic cells, *Rgs1^-/-^* cells are enabled to reconstitute the intestinal T cell compartments, we next generated congenic *Rgs1^-/-^* and *Rgs1^+/+^* mixed bone marrow chimeras as shown in **Figure 2A**. Sixty days post-reconstitution, the frequencies of T cell subsets from the small intestinal epithelium and lamina propria, and mLN and spleen were compared. Under these homeostatic conditions, the genetic deletion of *Rgs1* did not impair the reconstitution of all T cell compartments, even in a competitive setting. Occasionally, *Rgs1^-/-^* T cells were even slightly more abundant than their *Rgs1* ^+/+^ counterparts, e.g. CD4^+^ TCRαβ T cells in spleen and mLN (**Figure 2B**). The frequency of CD69^+^ CD103^+^ T_RM_ cells was identical on day 60 post-reconstitution in *Rgs1^+/+^* and *Rgs1^-/-^* CD8αβ^+^ (CD4^-^) TCRαβ IEL and LPL, and in *Rgs1^+/+^* and *Rgs1^-/-^* CD4^+^ (CD8αβ^-^) TCRαβ LPL. To define whether the deletion of *Rgs1* in *Rgs1* ^-/-^ T cells is compensated by other members of the Rgs gene family, we assessed the transcription profile of the other T-cell associated members of this gene family in CD8αβ+TCRαβ small intestinal IEL in *Rgs1^+/+^*, *vs*. *Rgs1^-/-^* mice under homeostatic conditions, and also in *Lm-*Ova (i.g.) infected mice, co-transferred with equal numbers of OT-I *Rgs1^+/+^* and *Rgs1^-/-^* cells. The deletion of *Rgs1*, however, did not affect the expression pattern of *Rgs*-2, 3, 9, 10, 14, 16, and 19 in tissue-associated small intestinal CD8αβ^+^ TCRαβ T cells from *Rgs1* ^-/-^ mice under homeostatic conditions. Following intestinal infection (i.g.) with *Lm-OVA* infection the absence of Rgs1 generally did not impact the expression pattern of the other Rgs family members early, and late after intestinal *Lm-*OVA infection, except for *Rgs10* which on day 8 post *Lm*-OVA infection was up-regulated in small intestinal OT-I *Rgs1* ^-/-^ IEL, but not in OT-I *Rgs1^-/-^* LPL (**Supplementary Figure 3D**). During the memory phase upon *Lm-*OVA infection (i.e. day 30 p.i.), *Rgs18* was slightly up-regulated in OT-I *Rgs1* ^-/-^ LPL (but not in IEL) (**Supplementary Figure 3**). Hence, only minor changes in the expression level of the other T cell-associated *Rgs* genes are seen in the presence, *vs*. absence, of *Rgs1* in T cells under homeostatic, and inflammatory conditions. Together with the reported distinct selectivity of Rgs family members for the different Galpha – subunits of GPCR (37) and their different intracellular distribution patterns (38), this makes it unlikely that changes in the expression of other Rgs gene family members may functionally compensate for the complete absence of Rgs1.

Conventional CD8^+^ T_RM_ cells in the intestine are commonly identified as TCRαβ CD8αβ^+^ T cells which co-express the putative residency markers CD69 and CD103 (5, 15). Since *Rgs1* expression strongly correlates with the expression of tissue residency signature genes (see **Figure 1E**), we next investigated whether the absence of *Rgs1* affects the tissue-resident phenotype of TCRαβ CD8αβ^+^ T cells from the small intestinal IEL and lamina propria lymphocytes (LPL) in bone marrow chimeras of *Rgs1* ^+/+^ and *Rgs1* ^-/-^ mice (**Figures 2C, D**). The percentage of CD69^+^ CD103^+^ CD4^+^ T cells and CD69^+^ CD103^+^ CD8αβ^+^ T cells in the small intestinal epithelium and lamina propria were comparable between *Rgs1^-/-^* and *Rgs1^+/+^* T cells in these bone marrow chimeras. These results indicate that under homeostatic conditions, the acquisition of a CD69^+^ CD103^+^ tissue-resident phenotype by CD4^+^ and CD8αβ^+^ T cells in the small intestine does not depend on *Rgs1* expression. Furthermore, not only the frequency of CD69^+^ CD103^+^ cells among CD4^+^ and CD8αβ^+^ TCRαβ cell subsets in the SI epithelium and lamina propria, but also the cell surface expression was comparable for Rgs1 sufficient, and deficient T cell subsets in the SI mucosa (**Figures 2C, D**).

### Rgs1 is induced in antigen-specific T cells at the site of the infection in response to microenvironmental cues

**Figure 3:**
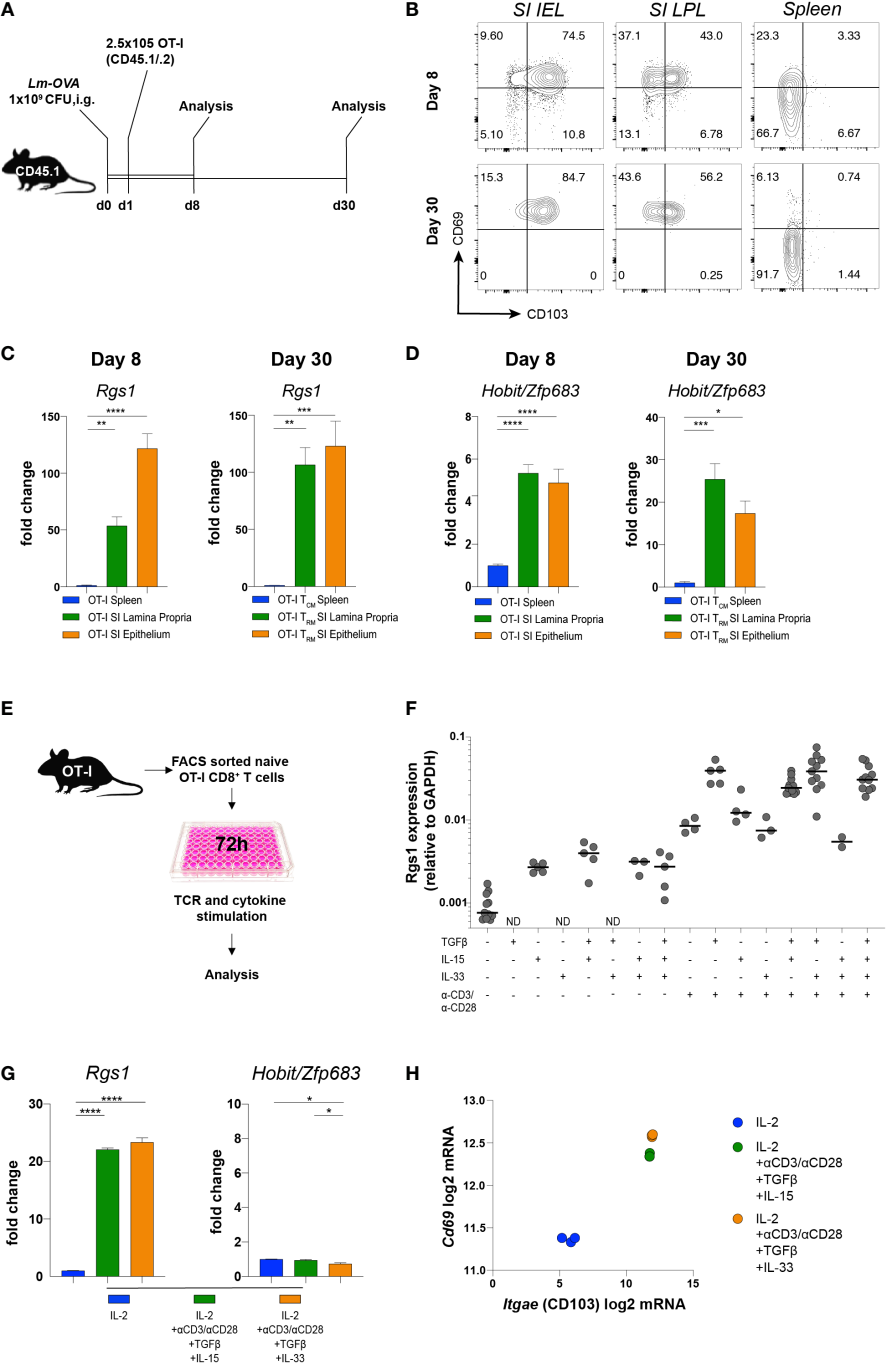
Induction of *Rgs1* mRNA expression in antigen-specific CD8^+^ T cells requires both TCR-specific activation and appropriate environmental cues. **(A)** Experimental set-up for **Figures 3B-D**. **(B)** Representative FACS plots showing CD69 and CD103 expression on OT-I cells at day 8 and 30 post gavage with Lm-OVA in small intestinal epithelium and l. propria, and spleen. **(C)** *Rgs1* expression and **(D)** *Hobit/Zfp683* expression in OT-I cells isolated and FACS sorted from the spleen, SI epithelium, and lamina propria on day 8 and 30 p.i. with *Lm-OVA*. (n=4-8, mean ± SEM, One-way ANOVA test followed by Tukey’s multiple comparison test, *, *p <*0.05; **, *p <*0.01; ***, *p <*0.001; ****, *p <*0.0001). **(E)** Experimental set-up to optimize Rgs1 mRNA-inducing conditions *in vitro*. **(F)** OT-I T cells from the spleen of naïve OT-I tg donor mice were FACS sorted and cultured for 72h in the presence or absence of αCD3/αCD28, TGFβ, IL-15, and/or IL33 as indicated. **(G)** *Rgs1*, *Hobit/Zfp683*, and **(H)** *Cd69* and *Itgae* mRNA induction in OT-I cells, cultured for 72 h under the indicated conditions. Fold change in the expression of *Rgs1* and *Zfp683*, mRNA upon *in vitro* culture *vs*. untreated *ex vivo* isolated, naïve splenic OT-I cells. (n=3, mean ± SEM, unpaired, One-way ANOVA test followed by Tukey’s multiple comparison test, *p <0.05; **p <0.01; ***p <0.001; ****p <0.0001). **(H)** *Cd69* and *Itgae* expression in OT-I T cells cultured in the presence of IL-2 alone, and under Rgs1 gene-inducing conditions as indicated. Experiments shown in **(G)** and **(H)** were performed twice with comparable results.

Transcriptional reprogramming of antigen-specific CD8^+^ T cells in non-lymphoid tissues is instrumental for local CD8^+^ T_RM_ cell differentiation. Intriguingly, this occurs at the early stage of the primary adaptive immune response in a microenvironment-dependent manner (39, 40). These differentiation events are also influenced by the affinity of the TCRαβ for the cognate antigen (41–43). To specifically assess the influence of the local microenvironment on *Rgs1* expression and, eventually, on potential *Rgs1*-mediated effects of CD8^+^ T cells differentiation, we used TCRαβ-transgenic, ovalbumin (OVA_257-264_) - specific OT-I CD8^+^ T cells (44) to monitor their expression of *Rgs1* during a primary immune response and the subsequent memory phase upon intragastric (i.g.) inoculation with ovalbumin - expressing *Listeria monocytogenes* (*Lm-OVA*). Specifically, OT-I T cells were transferred into CD45.1 congenic recipient mice, previously infected with OVA*-*expressing *Lm-OVA* (1x10^9^ CFU i.g./animal). Mice were analyzed at day 8 p.i., i.e. at a time when *Lm* are largely cleared in immunocompetent mice (45), and during the memory phase at day 30 post-infection (**Figure 3A**). Flow cytometric analyses of OT-I cells isolated from the small intestinal epithelium and lamina propria (but not from the spleen) revealed that a large proportion of antigen-specific OT-I cells expressed the T_RM_ cell-associated surface proteins CD69 and CD103 already by day 8 p.i. (**Figure 3B**). The percentage of these CD69^+^ CD103^+^ OT-I T_RM_ cells in the small intestine only marginally increased during the memory phase (day 30 p.i.). We then investigated the *Rgs*1 expression in antigen-specific OT-I cells by day 8 p.i. and during the memory phase (day 30 p.i.). Already by day 8 p.i. small intestinal lamina propria and intraepithelial OT-l T_RM_ cells showed a 50-, and a 130- fold increased *Rgs1* expression, respectively, when compared to splenic OT-I cells (**Figure 3C**). This *Rgs1* expression profile remained at this high level during the memory phase. In OT-l T_RM_ cells in the intestinal lamina propria, the already prominent *Rgs1* mRNA expression further increased two-fold between day 8 and day 30 p.i., while it remained low in CD62L^-^ CD44^+^ OT-I T_CM_ cells in the spleen (**Figure 3C**).

*Hobit/Zfp683* is a key transcription factor driving CD8^+^ T_RM_ cell differentiation at the site of infection (14, 46). Therefore, we analyzed the expression profile of *Hobit/Zfp683* in small intestinal IEL and LPL, as well as in splenic OT-I cells at day 8 and day 30 p.i. with *Lm-OVA*. When compared to splenic OT-I T_CM_ cells*, Hobit/Zfp683* mRNA expression of small intestinal OT-I IEL and LPL was 4- to 5-fold increased during the initial immune response (i.e., day 8 p.i.) (**Figure 3D**). During the subsequent memory phase (day 30 p.i.) *Hobit/Zfp683* mRNA expression in small intestinal OT-I IEL and OT-I LPL was further increased up to 15-20-fold in comparison to splenic OT-I T_CM_ cells (**Figure 3D**). This expression profile thus implies that similar to *Hobit/Zfp683* gene expression, the induction of *Rgs1* expression represents an early event during the local differentiation of CD8^+^ T_RM_ cells at the site of the infection. Upon infection, the further CD8^+^ T_RM_ cell differentiation is largely controlled by the tissue microenvironment (40, 47, 48). Accordingly, microenvironmental cues such as TGFβ, IL-15, or IL-33 were previously found to provide essential signals for local CD8^+^ T_RM_ cell differentiation in non-lymphoid tissues (47, 48). Hence, we hypothesized that *Rgs1* expression is induced by similar cues to drive local CD8^+^ T_RM_ cell differentiation in non-lymphoid tissues. For this purpose, OT-I cells from naïve donors were FACS purified, *in vitro* activated with anti-CD3ε/anti-CD28 mAbs, and further stimulated for 72 hours with various combinations of candidate cytokines in the absence, or presence, of TCR activation (**Figure 3E**). Under these *in vitro* culture conditions *Rgs1* expression was equally upregulated (**Figures 3F, G**), while intriguingly expression of *Hobit/Zfp683* mRNA was not induced (**Figure 3G**), thus indicating a differential transcriptional control of *Hobit/Zfp683* and *Rgs1* gene expression. This notion is further supported by the observation that mouse *Hobit^-/-^, and Hobit^-/-/^Blimp1^-/-^* CD8^+^ T_RM_ cells do not display altered *Rgs1* expression levels (**Supplementary Figure 4**) (14). The canonical T_RM_ cell signature genes encoding CD69, and CD103, however, were readily induced in anti-CD3/28 activated OT-I cells in the presence of TGFβ, combined with either IL15 or IL33 (**Figure 3H**).

Collectively, these findings reveal that the rapid *Rgs1* upregulation in antigen-specific CD8^+^ T cells represents an early event during local CD8^+^ T_RM_ cell differentiation at the site of infection. Moreover, this upregulation of *Rgs1* occurs in a *Hobit/Zfp683* independent manner and is largely controlled by local microenvironmental cues and CD3/TCRαβ activation.

### Rgs1 regulates the accumulation of antigen-specific CD8^+^ T cells in the small intestinal mucosa upon infection with Listeria monocytogenes-OVA

**Figure 4:**
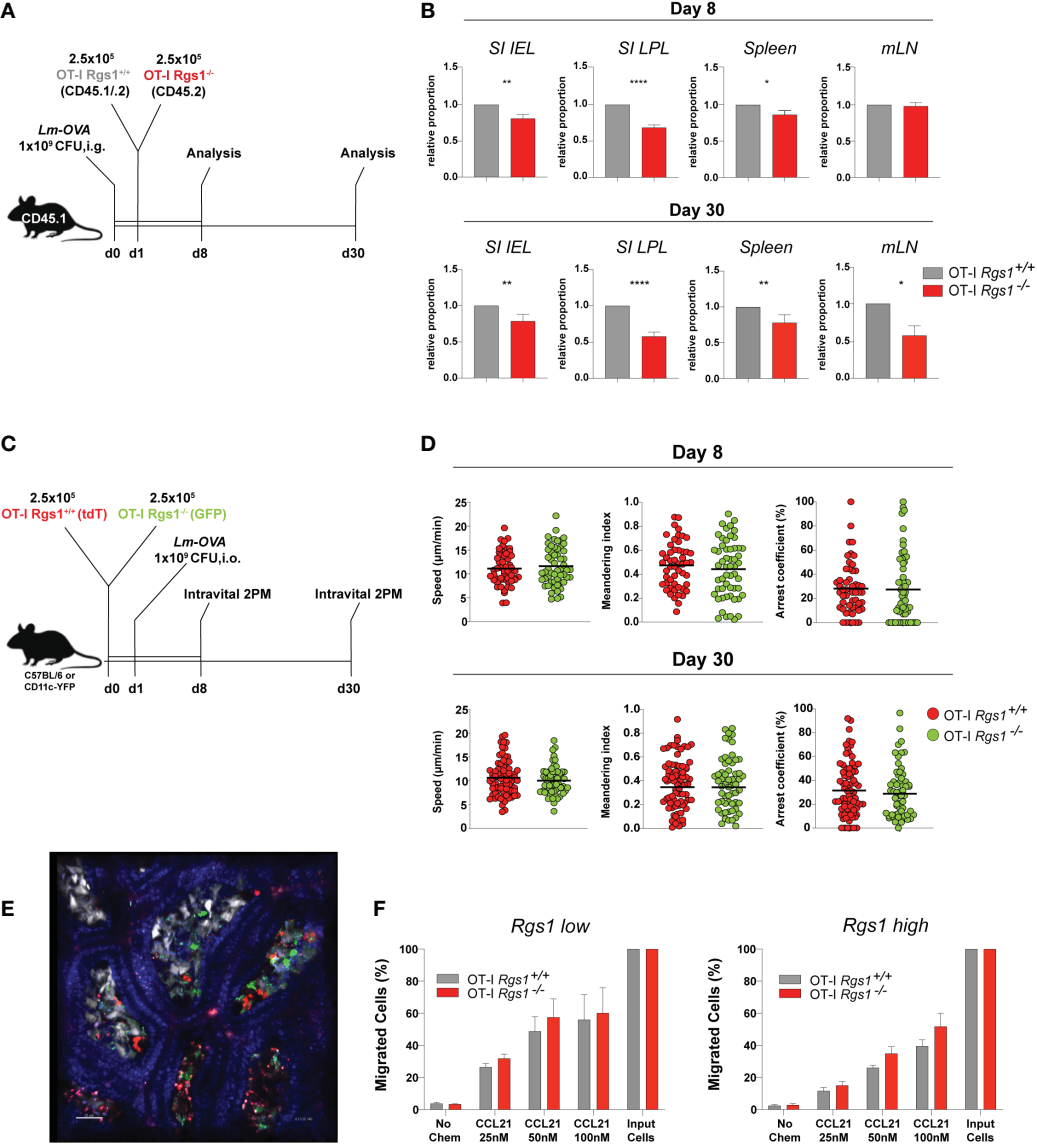
*Rgs1* promotes the efficient accumulation of effector CD8^+^ T cells and the early differentiation into CD8^+^ T_RM_ cells in the small intestine following oral *Lm-OVA* infection. **(A)** Experimental set-up for determining the relative frequencies of OT-I *Rgs1^+/+^ vs*. OT-I *Rgs1^-/-^* cells following *Lm-OVA* infection. **(B)** Pair-wise normalized ratios of OT-I *Rgs1^-/-^*: OT-I *Rgs1^+/+^* cell frequencies on day 8 p.i. (n=26, pooled from 5 independent experiments), and on day 30 p.i. (n=52-55, pooled from 6 independent experiments, mean ± SEM). **(C)** Rgs1-deficient and -sufficient OT-I CD8 T_RM_ cells display a similar patrolling behavior on days 8 and 30 p.i. with *Lm-OVA*. Experimental set-up for intravital 2-photon microscopy: CD45.1 mice were inoculated i.g. with 1x10^9^ CFU Lm-OVA. The following day a total of 5x10^5^ OT-I *Rgs1* ^+/+^ (tdT) and OT-I *Rgs1* ^-/-^ (GFP) cells (1:1) were transferred i.v. into the congenic recipient mice. On day 8 and 30 p.i. with *Lm-OVA*, groups of immune mice were anesthetized, and the SI was exposed for intravital 2-photon microscopy as described in Materials and Methods to assess the motility of OT-I *Rgs1* ^+/+^ (tdT) and OT-I *Rgs1^-/-^* (GFP). **(D)** Quantification of the assessed parameters (i.e. mean track speed, meandering index, arrest coefficient). Data pooled from 2 independent experiments (each dot represents a single cell track; grey=OT-I *Rgs1* ^+/+^, red=OT-I *Rgs1* ^-/-^; n=59-76), Mann-Whitney test, *p <0.05; **p <0.01; ****p <0.0001). **(E)** Representative image of the small intestinal villi of a CD11c-YFP recipient mouse of green (GFP) OT-I *Rgs1* ^-/-^, and red (tdT) OT-I *Rgs1* ^-/-^ cells on day 8 post-infection with *Lm-*OVA; in blue (Hoechst dye) epithelial cell nuclei (see Movies S1, S2 for day 8, and day 30 p.i. with *Lm-*OVA). **(F)** Percentage of migrated OT-I cells in a transwell chemotaxis assay in the absence, or presence, of the CCR7 agonist CCL21. OT-I cells were cultured in the presence of IL-2 only (non-Rgs1 inducing conditions, “*Rgs1 low*”); or activated with anti-CD3/CD28 mAb’s in the presence of the indicated cytokines for optimal *Rgs1* induction, (“*Rgs1 high*”) (see **Supplementary Figure 7** for details).

Having established that *Rgs1* expression is rapidly induced in antigen-specific CD8^+^ T cells at the site of infection, we next investigated whether Rgs1 expression in antigen-specific CD8^+^ T cells affects their accumulation during a local immune response against a pathogen. Therefore, OT-I *Rgs1^+/+^*, and OT-I *Rgs1^-/-^* cells were co-transferred into C57BL6/JRj mice, previously infected with *Lm-OVA* (i.g.). This congenic co-transfer system allows the direct comparison of the two genetically different OT-I cell populations and is not influenced by different efficiencies during cell isolation of individual mice or differences in the extent of infection. The relative frequency of wild type and OT-I *Rgs1^-/-^* cells were analyzed at day 8 p.i., i.e. at the time when in immunocompetent mice *Lm*-OVA is largely eliminated in the intestinal mucosa (45) and during the memory phase at day 30 p.i. (**Figures 4A, B**). At day 8 p.i. with *Lm-*OV*A* the frequencies of OT-I *Rgs1^+/+^* and OT-I *Rgs1^-/-^* cells were comparable in the draining mLN whereas in the spleen OT-I *Rgs1^-/-^* cells were slightly reduced in comparison to OT-I *Rgs-1* ^+/+^ cells. In comparison to wild-type OT-I cells, OT-I *Rgs1^-/-^* cells were substantially underrepresented in the SI IEL and, particularly, in the SI LPL at d8 p.i. (reduced by approximately 30%), while the difference was less prominent in the spleen (OT-1 *Rgs1* ^-/-^ cells reduced by approximately 15%) and in mLN where similar frequencies of OT-I *Rgs1^+/+^* and OT-I *Rgs1^-/-^* cells were observed. During the memory phase, i.e. at d30 p.i. the underrepresentation of OT-I *Rgs1* ^-/-^ cells was still most prominent in SI LPL, where approximately 40% more OT-I *Rgs1* ^+/+^ cells were detected. Also in the other T cell compartments analyzed (SI epithelium, spleen, and mLN) OT-I *Rgs1* ^+/+^ cells outnumbered their *Rgs1* ^-/-^ counterpart by 20 to 37% at d30 p.i. (**Figure 4B**). The underrepresentation of intestinal OT-I *Rgs1* ^-/-^ cells was also seen following a systemic infection of mice with LCMV-OVA where at day 8 post infection (i.p.) with *LCMV-*OVA; the OT-I *Rgs1* ^-/-^ cells were present in significantly lower frequencies in the small intestinal IEL and LPL compartment (**Supplementary Figure 5**). Although OT-I *Rgs1* ^-/-^ cells were significantly outnumbered by OT-I *Rgs1* ^+/+^ T cells on day 30 p.i, with *Lm*-OVA, no difference in the surface expression levels of the canonical CD8^+^ T_RM_ cell markers CD69, CD103, and CD44 was found between OT-I *Rgs1^-/-^* and OT-I *Rgs1* ^+/+^ cells in the small intestinal IEL and LPL compartment (**Supplementary Figure 6**).

Rgs1 has been previously identified as a critical regulator of the chemotactic activity of different immune cells including myeloid cells (49), B lymphocyte entrance into, and migration within, lymph nodes (50), migration and frequency of follicular helper T cells (51) and tumor-infiltrating T cells (52). Hence, given the observed underrepresentation of *Rgs1* ^-/-^ OT-I T cells in the small intestinal l. propria already at day 8 p.i. we hypothesized that *Rgs1* might impact the motility and patrolling of small intestinal CD8^+^ T cells (OT-I cells) in mice infected with *Lm-OVA*. To directly address this, OT-I *Rgs1^+/+^* cells, expressing tdTomato (tdT), and OT-I *Rgs1^-/-^ cells*, expressing green fluorescent protein (GFP), were co-transferred into recipient mice, which were infected p.o. with *Lm-*OVA one day later (**Figure 4C**). On day 8, and day 30 p.i., mice were anesthetized, and the small intestine mucosa was exposed for intravital 2-PM as described in *Materials and Methods.* Both OT-I *Rgs1* ^+/+^ and OT-I *Rgs1* ^-/-^ were motile throughout the intestinal villi. Intriguingly, we did not observe differences in mean track speed (average speed of a cell over length of imaging, µm/min), arrest coefficient (percentage of track segments with speeds <5µm/min), and meandering index (a measure of the deviation from a straight line of a migratory cell calculated as total displacement/path length of a cell track with a value of 1 indicating that the track is a straight line) between OT-I *Rgs1* ^-/-^ cells and OT-I *Rgs1* ^-/+^ cells (**Figures 4D, E**). Hence, we concluded that Rgs1 is not involved in the regulation of patrolling and motility of antigen-specific OT-I cells early, and late, after intestinal inflammation with *Lm*-OVA. Next, we assessed whether Rgs1 regulates the chemotactic migration of intestinal OT-I cells. To address this, we first determined the expression pattern of relevant chemokine receptors that may enable the emigration of antigen-specific CD8 T cells out of the intestinal mucosa and draining lymph nodes. As shown in **Supplementary Figure 7**, CCR7 which can mediate emigration of T cells out of mucosal sites (32) and which is desensitized by the activity of Rgs1 (51) is upregulated in naïve OT-I T cells upon anti-CD3/28 activation under *Rgs1*-inducing conditions. When activated OT-I *Rgs1* ^+/+^ *vs*. OT-I *Rgs1^-/-^* cells were assayed for their chemotactic migration towards CCR7 ligand CCL21/exodus-2 in a transwell assay, OT-I cells migrated in a dose-dependent manner along the CCL21 gradient. A trend for higher chemotactic activity was found in OT-I *Rgs1^-/-^* cells when higher CCL21 concentrations were used (**Figure 4E**). Differences in the chemotactic migration between OT-I *Rgs1* ^+/+^ and OT-I *Rgs1* ^-/-^ cells, however, were statistically not significant, even when the percentages of migrated cells were calculated (**Figure 4F**; **Supplementary Figure 7D**) Furthermore, also no difference in the chemotactic activity between OT-I *Rgs1^+/+^* and OT-I *Rgs1^-/-^* was found when the chemokines Ccl19 and Ccl25, but also the bioactive lipid mediator sphingosine-1-phosphate (S1P) were used as chemoattractants (data not shown). The deficiency for *Rgs1* did not affect the expression of chemokine receptors as shown for CCR9 where on day 8 p.i. with *Lm-*OVA identical frequencies of OT-I cells expressing CCR9 on their surface were observed (data not shown). Collectively, these findings thus indicate that differential chemotactic migration and mobility of OT-I cells in the presence, or absence, of *Rgs1*, are unlikely to solely explain the lower frequency of OT-I *Rgs*1^-/-^ cells in the small intestinal mucosa in mice early after infection with *Lm-*OVA.

**Figure 5:**
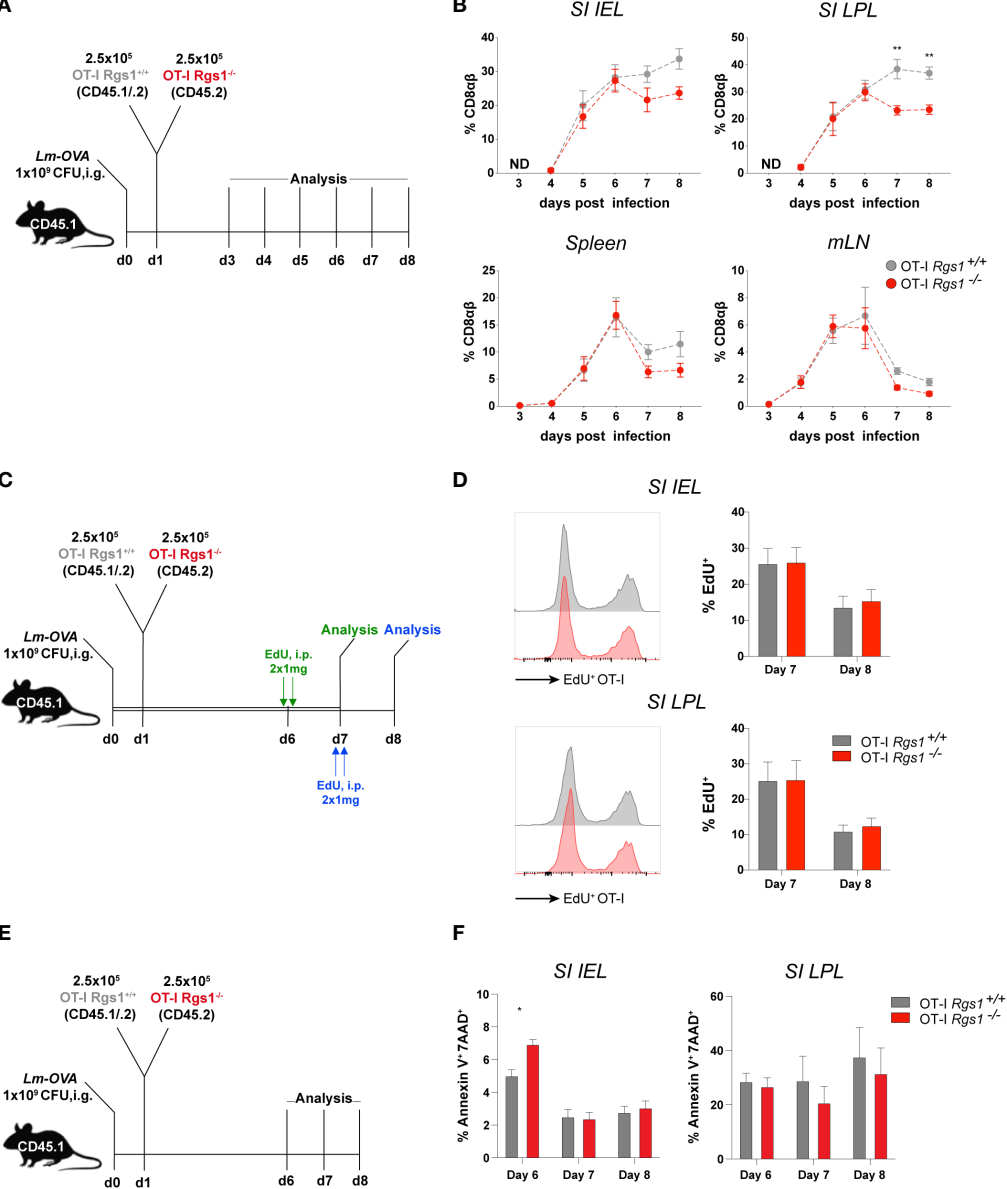
Antigen-specific OT-I *Rgs1^-/-^* T cells become underrepresented early after intestinal infection with *Lm-*OVA. **(A)** Experimental set-up to determine the relative frequency of co-transferred *Rgs1* ^+/+^ *vs*. *Rgs1^-^* ^/-^ OT-I CD8 T cells. **(B)** Frequencies of co-transferred OT-I *Rgs1^+/+^ vs*. OT-I *Rgs1^-/-^* T cells in the small intestinal epithelium (SI IEL), lamina propria (SI LPL), the spleen, and mesenteric LN (mLN), on day 3, 4, 5, 6, 7 and 8 after oral infection with *Lm-OVA*. (n=4-10, mean ± SEM, 2-way-ANOVA with Sidak’s multiple comparisons test, **, *p <*0.01). (ND, No transferred OT-I cells detected). **(C)** Experimental set-up for **D**. **(D)** Representative FACS histograms and respective frequencies of EdU positive proliferating *Rgs1^+/+^* OT-I (grey) and *Rgs1^-/-^* OT-I (red) cells from the small intestinal epithelium and lamina propria (n=6 at day 7 and n=8 at day 8, pooled from 2 independent experiments, mean ± SEM, 2-way-ANOVA with Sidak’s multiple comparisons test, all *p >*0.05 (not significant). **(E)** Experimental design to determine apoptosis rates of transferred OT-I cells. Apoptotic cells were detected by annexin V and 7-AAD staining in *Lm-OVA* infected (i.g.) recipient mice on days 6, 7 and 8 p.i. Mice were treated i.p. with 50 μg anti-ARTC2 nanobodies 20 min before sacrifice of the mice to prevent extracellular ATP/NAD^+^-induced, P2XR7/ARTC2-mediated apoptosis during isolation (29). **(F)** Frequencies of Annexin-V^+^ 7-AAD^+^ *Rgs1^+/+^* OT-I (grey) and *Rgs1^-/-^* OT-I (red) cells, representing late apoptotic from cells, from the small intestinal epithelium and lamina propria (n=4 at day 6, n=5 at day 7 and n=6 at day 8, pooled from 2 independent experiments, mean ± SEM, 2-way-ANOVA with Sidak’s multiple comparisons test); *, *p <*0.05.

To assess whether Rgs1 is involved in the early expansion of T cells at the site of infection, we first assessed the kinetics of OT-I *Rgs1* ^+/+^ *vs*. OT-I *Rgs1* ^-/-^ cells early after i.g. infection with *Lm-OVA*; i. e. starting at d3 p.i. when first OT-I T cells were detected, up to day 8 post-infection (**Figure 5A**). Up to day 6 p.i. with *Lm-OVA* the frequency of OT-I *Rgs1* ^+/+^ and OT-I *Rgs1* ^-/-^ cells in the small intestinal mucosa, and mLN and spleen were identical, and only after day 6 p.i. OT-I *Rgs1* ^-/-^ T cells became rapidly underrepresented (**Figure 5B**). Intriguingly, the preferential disappearance of OT-1 *Rgs1^-/-^* cells, particularly in the small intestinal mucosa between day 6 and 8 p.i., is seen in all individual mice analyzed on day 7 and 8 p.i. (**Supplementary Figure 8**). From day 8 p.i. on, however, the ratio of OT-I *Rgs1* ^+/+^ to OT-I *Rgs1* ^-/-^ OT-I cells in the small intestinal T cell compartments remained rather constant, indicating that the selective advantage of OT-I *Rgs1* ^+/+^ cells is likely associated with the initial expansion and/or the contraction of antigen-specific CD8 T cells at these sites. Therefore, we next determined the proliferative capacity of OT-I *Rgs1* ^+/+^ *vs*. *Rgs1* ^-/-^ cells, isolated at day 7 and day 8 p.i. after a 24h labeling period with the click chemistry-based EdU assay (26, 27) (**Figure 5C**). As shown in **Figure 5D** on both day 7, and day 8 p.i., OT-I *Rgs1* ^+/+^, and *Rgs1* ^-/-^ cells showed identical proliferation rates. Hence, the reduced expansion of OT-I *Rgs1* ^-/-^ cells observed from day 6 p.i. onwards cannot be solely explained by a reduced proliferative capacity of OT-I *Rgs1* ^-/-^ cells. Staining of isolated small intestinal OT-I cells on days 6, 7, and 8 p.i. for Annexin V and 7-AAD, however, revealed a significantly higher apoptosis rate in OT-I *Rgs1* ^+/+^ *vs*. OT-I *Rgs1* ^-/-^ IEL on day 6 p.i. in the small intestinal mucosa, while in the LPL the percentage of 7-AAD^+/^Annexin V^+^ cells remained equally high in the OT-I *Rgs1* ^+/+^ *vs*. OT-I *Rgs1* ^-/-^ LPL, isolated between day 6 and 8 p.i. despite the administration of anti-ARTC2 nanobodies prior to cell isolation (**Figure 5F**).

The T-box transcription factors T-bet (*Tbx21*), and Eomes were previously reported to reciprocally control memory precursor effector cells (Eomes), and short-lived effector cells (SLEC) (T-bet) differentiation, respectively (6, 42, 53). Hence, we next compared the *Tbx21/Eomes* mRNA expression ratio in FACS purified wild type and OT-I *Rgs1^-/-^* cells, isolated from the spleen and the small intestine at day 8 p.i. with *Lm-OVA*. Indeed, we observed a slight increase in the *Tbx21/Eomes* ratio in OT-I *Rgs1^-/-^* compared to OT-I *Rgs1^+/+^* cells, isolated from the small intestine (IEL and LPL) (**Supplementary Figure 9A**), whereas in the spleen no signs for an induction of *Rgs1* expression, or an altered *Tbx21*:*Eomes* ratio is seen. Hence, lower *Tbx21: Eomes* ratios are associated with strong induction of *Rgs1* expression in the OT-I *Rgs1^+/+^* cells. This may indicate a higher propensity of intestinal OT-I *Rgs1* ^-/-^ cells to differentiate into SLEC at the site of the infection, which may contribute to the observed preferential disappearance of OT-I Rgs1^-/-^ cells from the small intestinal mucosa (**Figure 5**). Unfortunately, we were unable to reliably assess the SLEC: MPEC ratio according to the differential expression of CD127 and KLRG1 since we realized that the addition of dithiothreitol (DTT) during the isolation of intestinal T cells resulted in a reduced staining for CD127 (54). The observation of a rather constant ratio of OT-I *Rgs1* ^-/-^: OT-I *Rgs1* ^+/+^ T cells between day 8, and day 30 p.i. with *Lm-*OVA, however, indicates that Rgs1 appears to be more critical for the early expansion and, in particular, the maintenance of antigen-specific CD8αβ+ TCRαβ T cells under inflammatory conditions.

To determine whether Rgs1 exerts also an effect on the functional differentiation of local T cells resulting in a distinct expression profile of core signature genes (4–6), we compared the expression profiles of CD8^+^ T_RM_ core signature genes of small intestinal CD69^+^ CD103^+^ OT-I *Rgs1^-/-^* and OT-I *Rgs1^+/+^* T_RM_ LPL at day 30 p.i. with *Lm-OVA*. Intriguingly, OT-I *Rgs1* ^+/+^ and *Rgs1^-/-^* OT-I T_RM_ cells displayed pronounced differences in the expression profile of several CD8^+^ T_RM_ core signature genes (**Supplementary Figure 9B**). Remarkably, several genes characteristically overexpressed in CD8^+^ T_RM_ cells and T_RM_ cell-mediated responses (e.g. *Itgae, Cd69*, *Tcf7, Xcl1*), were significantly up-regulated in OT-I *Rgs1* ^+/+^ cells on d30 p.i. in small intestinal LPL, whereas other genes like *Sipr1* and *Cmah*, characteristically down-regulated in T_RM_ T cells (14, 55), were expressed at significantly lower levels in OT-I *Rgs1^+/+^* cells when compared to OT-I *Rgs1^-/-^* cells.

scRNA sequencing experiments revealed the functional heterogeneity of tissue-resident CD8αβ^+^ T cells (56) with a resident subpopulation of Blimp1^hi^ Klrg1^hi/int^ CD127 small intestinal IEL which dominate early after systemic LCMV (i.p) infection to become tissue-resident effector memory cells, whereas Id3^+^ KLRg1^lo^ CD127^hi^ cells represented tissue-resident memory precursor cells, which later become tissue-resident memory cells. When we analysed those genes differentially expressed in tissue-resident effector cells *vs*. tissue-resident memory cells (56), some of those genes preferentially expressed in resident effector IEL (“effector gene signature”), notably, *Prdm1/Blimp1*, *Icos*, and *Gzmb* were indeed more prominently expressed in *Rgs1^-/-^* OT-I IEL, isolated on day 8 post-infection with *Lm*-OVA (data not shown). This indicates indeed that in T cells Rgs1 might favor and support the acquisition and maintenance of a T_RM_ cell signature rather than their differentiation into effector-like tissue-resident cell populations.

### Genetic deletion of Rgs1 impairs CD8^+^ T_RM_ cell-mediated protection from systemic dissemination of L. monocytogenes-OVA during reinfection

**Figure 6:**
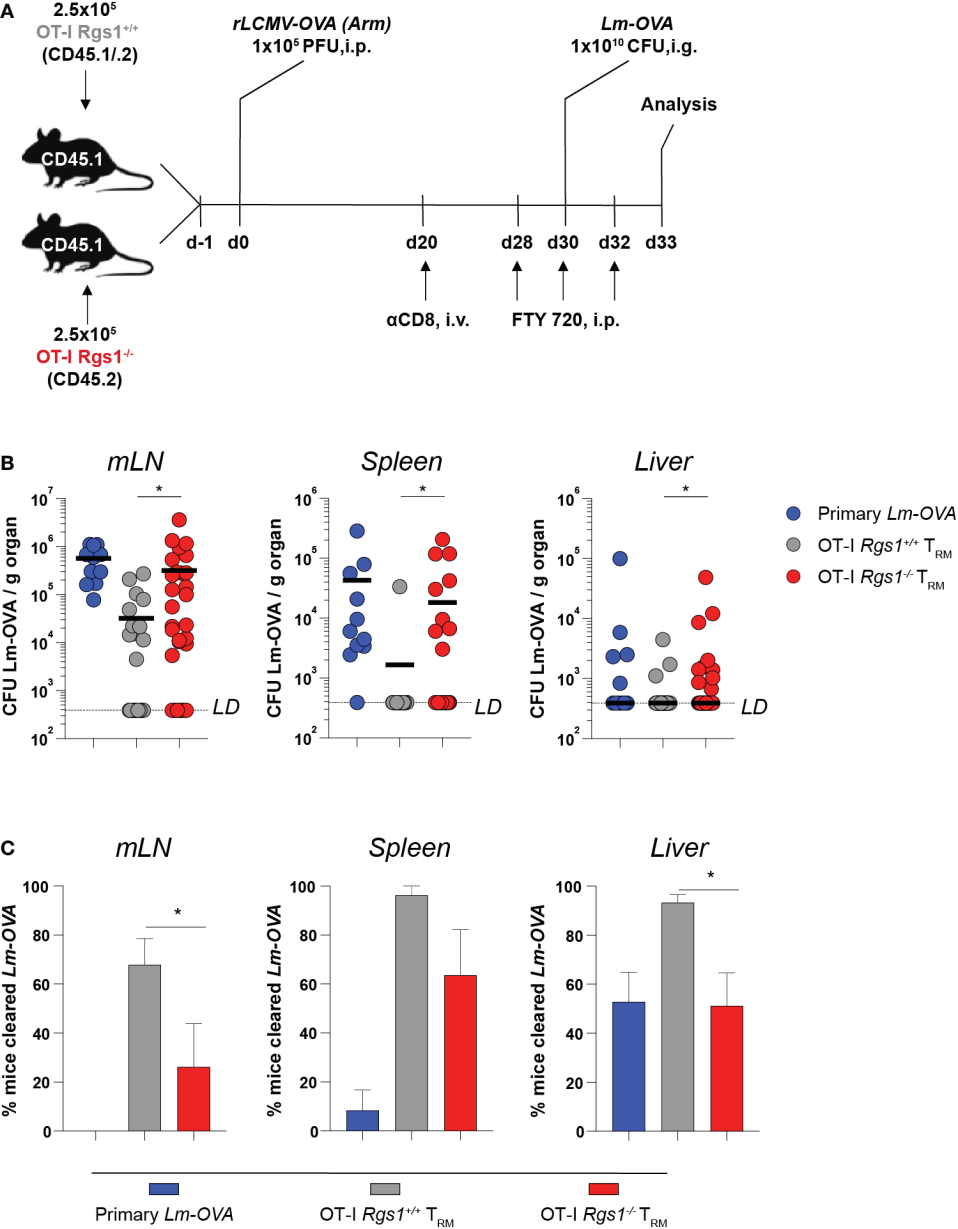
*Rgs1*-deficient CD8^+^ T_RM_ cells in the small intestine confer impaired protection from systemic pathogen dissemination upon local infection with *Lm-OVA*. **(A)** Experimental set-up. **(B)** *Lm-OVA* titers, measured on day 3 post re-challenge in the mLN, spleen, and liver of control mice without transferred OT-I T cells and without rLCMV-OVA infection on d0, but infection with *Lm-*OVA on d30 (“primary *Lm-OVA*”, blue, n=11); mice with intestinal wild type OT-I T_RM_ cells (“OT-I *Rgs1* ^+/+^ T_RM_”, grey, n=25-28) and mice with intestinal *Rgs1^-/-^* OT-I T_RM_ cells (“OT-I *Rgs1^-/-^* T_RM_”, red, n=30-31). Titers of the OT-I *Rgs1^-/-^* T_RM_ group were compared to the OT-I *Rgs1* ^+/+^ T_RM_ group (individual values + mean, LD (limit of detection was calculated as the hypothetical smallest detectable *Lm-OVA* colony count (=1) in the largest collected piece of organ), unpaired Mann-Whitney *t*-test, two-tailed, *p <0.05. **(C)** Percentage of mice in the “primary *Lm-OVA*”, “OT-I T_RM_” and “OT-I T_RM_ *Rgs1^-/-^*” groups with non-detectable *Lm-OVA* (Lm cleared) in the indicated organs. Means of 3 independent experiments (each with n=7-13 per group) between the “OT-I T_RM_” and “OT-I *Rgs1^-/^* ^-^ T_RM_” groups were compared (mean ± SEM, unpaired Student´s *t*-test, two-tailed, *p < 0.05).

The establishment of an immediate recall response to achieve rapid elimination of re-invading pathogens is the functional hallmark of CD8^+^ T_RM_ cells (5, 57, 58). Hence, we examined the protective capacity of OT-I *Rgs1^-/-^ vs*. wild-type OT-I T_RM_ cells upon a local re-infection of the small intestine. To this end, we established a heterologous infection protocol as depicted in **Figure 6A**. Recipient mice were administered either 5x10^5^ OT-I *Rgs1^+/+^* or OT-I *Rgs1^-/-^* cells (i.v.) and were infected with 1x10^5^ PFU r*LCMV-OVA* i.p. 24h later. At day 30 post-infection, the mice were re-challenged with 1x10^10^ CFU *Lm-OVA* (i.g.). To specifically compare local CD8^+^ T_RM_ cell-mediated immunity between mice harboring tissue-resident OT-I *Rgs1^+/+^* and OT-I *Rgs1^-/-^* T_RM_ cells in the small intestine, the circulating CD8^+^ T cells, including circulating OT-I *Rgs1^+/+^* and OT-I *Rgs1^-/-^* memory cells, were depleted by injecting the mice with anti-CD8α mAb (450μg/animal, i.p.) before the secondary infection (59). This allowed us to specifically assess the impact of Rgs1 on the immunoprotective potential of local tissue-resident antigen-specific T cells. To prevent the subsequent S1P-receptor-dependent T cell egress of effector OT-I cells (60, 61), which may also contain Rgs1 expressing effector cells (56) from secondary lymphoid organs to the site of intestinal infection, the sphingosine1-phosphate receptor analogue FTY720 (Fingolimod, 25μg/mouse) was administered i.p. during the re-challenge period (at day 28, day 30, and day 32 post primary infection) (**Figure 6A**). At day 3 post rechallenge, the extent of OT-I *Rgs1^+/+^ vs*. OT-I *Rgs1^-/-^* T_RM_ cell-mediated protective immunity was compared by determining extra-intestinal dissemination of *Lm-OVA* to the mLN, the spleen, and the liver (**Figure 6B**). Previously immunized mice harboring OT-I *Rgs1^+/+^* T_RM_ cells at the site of the infection showed markedly decreased *Lm-OVA* titers in mLN, spleen, and liver (**Figure 6B**). Strikingly, mice harboring intestinal *Rgs1^-/-^* OT-I T_RM_ cells displayed significantly higher *Lm-OVA* titers in mLN, spleen, and liver than those with OT-I *Rgs1^+/+^* T_RM_ cells, thus, demonstrating that the absence of *Rgs1* in intestinal CD8^+^ T_RM_ cells diminishes their capacity to control the systemic spreading of *Lm-OVA* upon local re-infection (**Figure 6B**). Accordingly, significantly fewer mice reconstituted with *Rgs1^-/-^* OT-I cells were able to completely prevent dissemination of *Lm-OVA* to the mLN and liver on day 3 post reinfection (i.e. no viable Lm-OVA in mLN and liver detected upon *ex vivo* culture) than those mice reconstituted with OT-I *Rgs1^+/+^* cells (**Figure 6C**). The lower *Lm-*OVA titers found in the spleen of rechallenged mice harboring OT-I *Rgs1^+^* ^/+^ cells may be attributed not only to the higher frequency of OT-I *Rgs1* ^+/+^ cells seen on day 30 p.i. with *Lm-*OVA (**Figure 4B**), but also to a more potent T cell response by OT-I *Rgs1* ^+/+^ cells at the site of intestinal infection as evidenced by a more pronounced IFNγ production in intestinal OT-I *Rgs1* ^+/+^ cells on day 2 and day 3 after OVA-specific intestinal rechallenge with *Lm*-OVA (**Supplementary Figure 10**), which - in contrast to recipients of OT-I *Rgs1^-/-^* cells - might thus prevent an excessive translocation and systemic dissemination of the pathogen from the intestinal lumen to the intestinal mucosa and to extraintestinal sites. These results thus demonstrate that the absence of *Rgs1* substantially impairs the protective capacity of the local intestinal CD8^+^ T_RM_ cells during local reinfection.

## Discussion

The local persistence of T_RM_ cells is critical for the successful T_RM_ cell-mediated protection of barrier tissues. The signature genes that are characteristically expressed in non-circulating CD8^+^ T_RM_ cells and which likely dictate their tissue residency also include distinct members of the *Rgs* gene family (12, 62). The comprehensive analyses of published data and our own results (**Figure 1**) confirmed that small intestinal tissue-resident TCRγδ and TCRαβ T cell subsets preferentially express *Rgs1* which distinguishes them from circulating, systemic T cell subsets (3, 11, 15, 41). Despite its prominent expression in T_RM_ cells, however, the impact of Rgs1 on the establishment of a local T cell-mediated memory has not been addressed so far. We now identify *Rgs1* as a critical factor in the efficient generation and maintenance of antigen-specific CD8^+^ T_RM_ cells as the genetic deficiency for *Rgs1* in antigen-specific CD8αβ^+^ T cells results in lower numbers of local antigen-specific T_RM_ CD8 T cells. Consequently, in comparison to their *Rgs1* ^+/+^ counterparts, intestinal antigen-specific *Rgs1* ^-/-^ CD8αβ^+^ T_RM_ cells show an impaired capacity to limit pathogen dissemination to extra-intestinal organs during local reinfection.

Under homeostatic conditions *Rgs1*-deficiency does not appear to have a major effect on the reconstitution of the T cell compartments in the SI mucosa, draining mLN and the spleen (**Figure 2**; **Supplementary Figure 2**). Genetic absence of Rgs1 did not substantially impact the expression profile of the other T cell expressed members of the Rgs gene family under homeostatic, and inflammatory conditions (**Supplementary Figure 3**). Furthermore, due to the reported distinct selectivity of Rgs family members for the different Galpha – subunits of GPCR (37), but also by their different intracellular distribution (38), it appears rather unlikely that up-regulation of other Rgs family members may functionally compensate for the complete absence of Rgs1.

One of the most compelling findings was the dramatic impact of Rgs1 deficiency on the numbers of antigen-specific OT-I cells recovered from small intestinal LPL after more than 6 days p.i. with *Lm*-OVA (**Figure 5B**), but also following the acute systemic infection with *LCMV*-OVA **Supplementary Figure 5**). The underrepresentation of OT-I *Rgs1* ^-/-^ cells at the site of infection cannot be fully attributed to differences in the proliferative activity of *Rgs1* ^+/+^ *vs*. *Rgs1* ^-/-^ OT-I cells during this period (**Figure 5D**). Intriguingly, the observed underrepresentation of OT-I *Rgs1* ^-/-^ cells in the small intestinal LPL after day 6 p.i. with Lm- OVA overlaps with the reported timing of the fate decision in CD8^+^ T cells to differentiate into either CD127^high^ KLRG1^low^ MPEC, or CD127^low^ KLRG1^high^ SLEC (63, 64) which also occurs early after initial antigen recognition by a CD8^+^ T cell, i.e. even before the peak of the primary T cell response against *Listeria monocytogenes* (2, 47, 65). The observed higher propensity of OT-I *Rgs1* ^-/-^ IEL to undergo apoptosis early (day 6) post-infection with *Lm-*OVA (**Figure 5F**) indicates that absence of Rgs1 may indeed critically regulate the fate decision of antigen-specific CD8^+^ T cells, particularly, during inflammatory conditions.

A well-established critical factor to decide the further fate of a T cell is the affinity of the TCR for the cognate antigen, with paradoxically, higher affinity TCR^+^ T cells being preferentially directed toward the SLEC pathway (41–43). Nevertheless, in our TCRαβ transgenic (tg) system (with a fixed affinity for the OVA_257-264_ peptide SIINFEKL), TCR affinity for the cognate antigen is unlikely to represent a fate-deciding factor, and, hence, other factors, including the spatial distribution of OT-I *Rgs1^+/+^ vs*. OT-I *Rgs1^-/-^* T cells in the small intestinal mucosa, might contribute to the observed preferential accumulation of OT-I *Rgs1^+/+^* T cells. In this context, it is remarkable that the *Tbx21*:*Eomes* ratio, measured in small intestinal intraepithelial and lamina propria OT-I cells, isolated on d8 p.i. is preferentially elevated in OT-I *Rgs1* ^-/-^ cells (**Supplementary Figure 9**), which may indicate their preferential further differentiation into SLEC cells (41, 42). At the same time (d8 p.i. with *Lm-*OVA i.g.)) a drastic induction of Rgs1 expression in OT-I *Rgs1* ^+/+^ cells is seen in the SI mucosa (IEL and LPL) (**Figure 3C**). This prominent *Rgs1* mRNA expression level in OT-I cells in the small intestinal IEL and LPL compartment of *Lm-*OVA infected mice was maintained throughout the entire infection period (up to day 30 p.i.) while the induction of *Hobit/Zfp683* was less pronounced, but also gradually increased during the memory phase of infection (day 30 p.i) (**Figures 3C, D**). This antigen-driven up-regulation of *Rgs1* in the transferred OT-I cells is likely supported by the local intestinal microenvironment since cytokines enriched in the small intestinal mucosa, such as TGFβ and IL15 markedly up-regulated *Rgs*1 expression in activated OT-I cells *in vitro* (**Figure 3G**). Under the same *in vitro* culture conditions, however, *Hobit/Zfp683* mRNA expression was not induced, indicating a distinct regulation and kinetics of *Hobit/Zfp368*, and *Rgs*1 gene expression (**Figure 3H**). Conversely, *Rgs*1 mRNA expression was also reported in bone marrow-derived T_RM_ cells from *Hobit/Zfp683^-/-^, and Hobit^-/-^ x Blimp1^-/-^* mice (14) (**Supplementary Figure 4**). Hence, the expression of *Rgs1* in CD8^+^ T cells is regulated independently from Hobit/Zfp683 and Blimp1/Prdm1. Despite this distinct regulation at the transcriptional level, however, Hobit and/or Blimp1, but also other transcription factors involved in the regulation of tissue residency of immune cells, such Id2, Id3 and Bhlhe40, may still interact functionally with Rgs1 *via* their regulated genes in a microenvironment-dependent manner.

The observed rapid TCRαβ-mediated *Rgs1* induction *in vitro* in T cells, preferentially, but not exclusively, in the presence of TGFβ, but also of other mediators such as IL15 and IL-33 (**Figure 3G**) suggests that cytokine-, and TCR activation-induced *Rgs1* expression may induce a state of unresponsiveness also to Gαi-dependent, G-protein coupled receptor (GPCR)-mediated signals, including the chemotactic recruitment of immune cells (10, 13, 19, 50). Our 2-photon microscopy analysis, however, did not reveal a substantial Rgs1-mediated effect on the patrolling of OT-I cells *in vivo* in the small intestinal mucosa (**Figure 4D**). Accordingly, some antigen-specific CD8^+^ T_RM_ cell subsets have been previously described to have a patrolling profile also in chemokine-independent situations (66). Furthermore, the promigratory effects by environmental chemokines reportedly manifests itself in only minor differences in directional persistence (9) which is rather difficult to measure by intravital microscopy in the complex architecture of the small intestine. Our extensive chemotaxis assays *in vitro* did not reveal direct evidence for a major impact of Rgs1 expression on chemotactic responses by antigen-specific CD8 T cells. Nevertheless, we cannot rule out that subtle differences in the chemotaxis of Rgs1 deficient *vs*. sufficient CD8αβ^+^ T cells (**Figure 4D**) may over time lead to substantial differences in the local persistence of Rgs1- deficient, *vs*. sufficient T cells, and hence, may also contribute to the observed underrepresentation of OT-I *Rgs1^-/-^* T cells also in spleen and mLN on day 30 p.i. with Lm-OVA (**Figure 4B**). Additional Rgs1-mediated effects on CD8 T cells, however, cannot be ruled out, particularly, since a recent structural analysis revealed a high selectivity of Rgs1 not only for Gαi but also for Gαq subunits (37). Indeed, Gαq GPCRs, including GPR43/FFAR2 (67) are known to be expressed by T cell subsets also in the intestinal mucosa (68).

At day 30 p.i. with *Lm-*OVA, OT-I *Rgs1^+/+^* and OT-I *Rgs1^-/-^* cells displayed an identical surface expression pattern of the common CD8^+^ T_RM_ markers (i.e. CD44, CD69 and CD103) (**Supplementary Figure 6**), hence, indicating that expression of these T_RM_ signature proteins is not strictly dependent on Rgs1 expression.

Rgs1 expression may not only affect the differentiation of a given T cell into SLEC *vs*. MPEC, but also modulate their functional properties. This notion is supported by the observed superior production of IFNγ by OT-I *Rgs1* ^+/+^ cells early during reinfection (**Supplementary Figure 10**), and the distinct expression levels of CD8^+^ T_RM_ core signature genes such as *Xcl1*, *Itgae*, *Inpp4b*, *S1pr1*, and CD69 in small intestinal *Rgs1^-/-^ vs*. *Rgs1^+/+^* OT-I T_RM_ cells in the lamina propria on d30 p.i. (**Supplementary Figure 9B**). The chemokine *Xcl1* mediates the selective recruitment of Xcr1^+^ CD8α^+^ cross-presenting dendritic cells and of Xcr1^+^ lymph node resident dendritic cells to the site of antigen priming (69). In a positive feedback loop, the recruitment of Xcr1^+^ CD8α^+^ dendritic cells can significantly enhance the cytotoxic capacity and IFNγ production of Xcl1-secreting CD8^+^ T cells (28, 70). (**Supplementary Figures 9B**, **10B**). This mechanism may thus further contribute to the observed superior control of *Lm-*OVA infection by *Rgs1^+/+^* OT-I cells.

Collectively, *Rgs1* expression by intestinal CD8^+^ T cells affects the accumulation of antigen-specific CD8^+^ T cells in the small intestinal mucosa following i.g. infection with *Lm-OVA*. Rgs1-deficient T cells in the small intestinal IEL compartment appear to be more prone to undergo apoptosis following antigen-specific priming (**Figure 5F**; **Supplementary Figure 8**) and may be even functionally impaired during a recall response (**Supplementary Figure 10**). This critically enhances their capacity to limit pathogen dissemination to extra-intestinal organs during local reinfection. While the underlying mechanisms are not fully understood, these results obtained with *Rgs1* deficient CD8^+^ T cells highlight the requirement of an increased *Rgs1* expression for the efficient accumulation and maintenance of antigen-specific CD8^+^ T cells at the site of the infection, but also in secondary lymphoid organs. The reduced accumulation of antigen-specific T cells of *Rgs1* ^-/-^ mice may be attributed in part to an impaired capacity of OT-I *Rgs*1^-/-^ cells to preferentially differentiate into functionally competent CD8^+^ MPEC in sufficient numbers.

Murine and human T_RM_ cells display a similar *Rgs1/RGS1* expression profile in distinct barrier tissues, including the intestinal mucosa, indicating a highly conserved function of this gene for local T_RM_ cell differentiation and function (**Figure 1**; **Supplementary Figure 1** (4, 15). It remains to be investigated whether the known genetic association between RGS1 SNP’s and mainly T cell-mediated pathologies including multiple sclerosis (71, 72), celiac disease (73, 74), and type 1 diabetes (73, 74), can be linked to aberrant T_RM_ cell formation, described here in the genetic absence of *Rgs1* in antigen-specific CD8 T cells. Our novel findings on the consequences of Rgs1-deficiency in T cells may open new avenues of further investigation into the different aspects of Rgs1 functions in T cell biology, particularly, in the early control of T cell expansion *vs*. contraction following antigen-specific activation of T cells at barrier tissues.

## Data availability statement

The original contributions presented in the study are included in the article/**Supplementary Material**, further inquiries can be directed to the corresponding author/s.

## Ethics statement

The animal study was reviewed and approved by Cantonal Veterinary Office Canton of Bern CH-3000 Bern Switzerland (license BE97/2020).

## Author contributions

DvW and BG performed most of the experimental work and wrote the first draft of the manuscript. JA, CC and DZ performed additional *in vivo* experiments, TG carried out in silico analysis, JS, MS, AH, BI, JK, DM, DF and GT revised the manuscript, JK and AH provided mouse lines, NP and DM produced and provided *Lm*-Ova. GT, DF and JA provided critical scientific input, RB and AC-F provided critical assistance in the *in vivo* and *in vitro* studies, NC and CM supervised the project and CM finalized the manuscript. All authors contributed to the article and approved the submitted version.

## Supplementary material

Supplementary Videos S1 and S2 CD45.1 mice were inoculated i.g. with 1x10^9^ CFU Lm-OVA. The following day a total of 5x10^5^ OT-I Rgs1^+/+^ (tdT) and OT-I Rgs1^-/-^ (GFP) cells (1:1) were transferred i.v. into the congenic recipient mice. On day 8 (Movie S1) and day 30 (Movie S2) post-infection with Lm-OVA, groups of immune mice were anesthetized, and the small intestine was exposed for intravital 2-photon microscopy as described in Materials and Methods to assess the motility of OT-I Rgs1^+/+^ (tdT) and OT-I Rgs1^-/-^ (GFP) cells.
