# Supplementary material for: Regulator of G-protein signaling 1 critically supports CD8+ TRM cell-mediated intestinal immunity
Source: Front Immunol. 2023 Apr 20;14:1085895. doi: 10.3389/fimmu.2023.1085895 (PMC10158727; doi:10.3389/fimmu.2023.1085895)
Supplement: Supplementary Videos S1 and S2 — CD45.1 mice were inoculated i.g. with 1x10^9^ CFU Lm-OVA. The following day a total of 5x10^5^ OT-I Rgs1+/+ (tdT) and OT-I Rgs1-/- (GFP) cells (1:1) were transferred i.v. into the congenic recipient mice. On day 8 (Movie S1) and day 30 (Movie S2) post-infection with Lm-OVA, groups of immune mice were anesthetized, and the small intestine was exposed for intravital 2-photon microscopy as described in Materials and Methods to assess the motility of OT-I Rgs1+/+ (tdT) and OT-I Rgs1-/- (GFP) cells. [file DataSheet_1.pdf]

Supplementary Figure 1

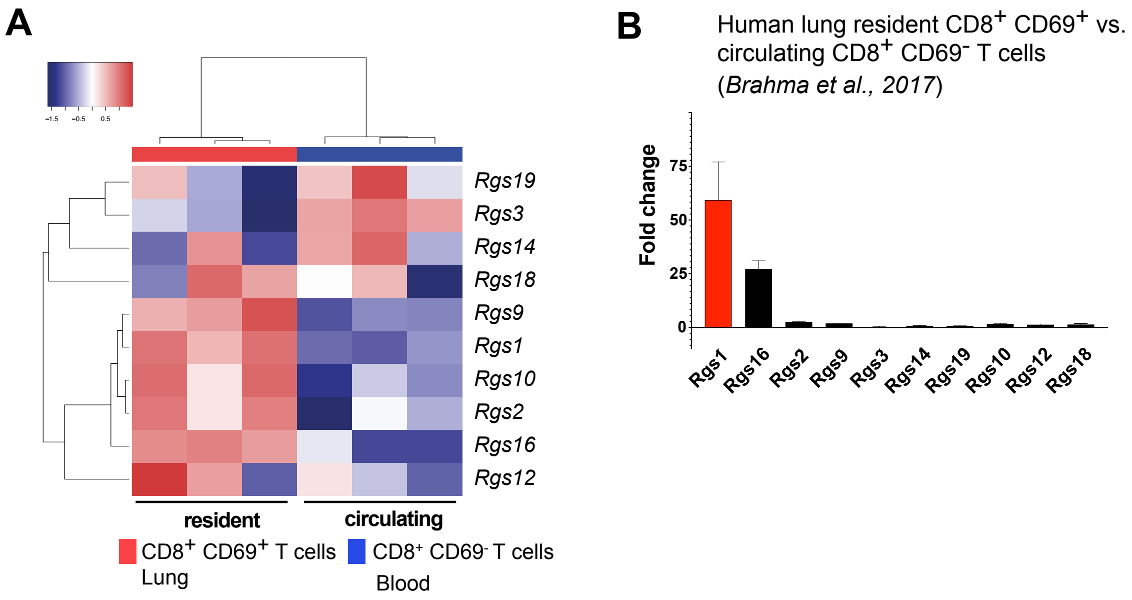

The RGS1 gene is preferentially expressed also in human CD8 T_{RM} cells

(A) Heat map showing human RGS gene expression profile of circulating ($CD69^-$) and resident ($CD69^+$) $CD8^+$ memory T cells (n=3 per indicated subset). **(B)** Fold change in the expression of RGS gene members in resident lung resident ($CD69^+$) vs. circulating blood ($CD69^-$) CD8 memory T cells (n=3). Data were retrieved from https://www.ncbi.nlm.nih.gov, GSE94964, Brahma et al., Cell Rep, 2017.

Supplementary Figure 2

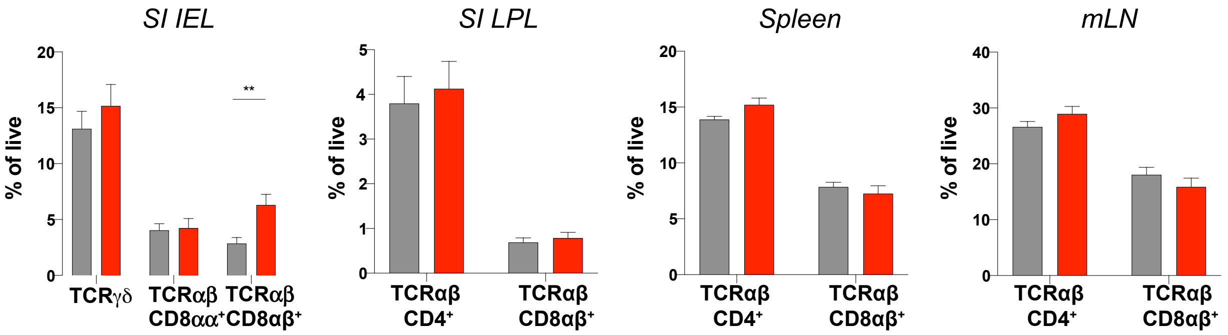

Rgs1- **deficiency does not affect the reconstitution of the T cell compartment under homeostatic conditions.**

The indicated T cell subsets were isolated from the SI epithelium (SI IEL) and lamina propria (SI LPL), the spleen and the mesenteric lymph nodes (mLN) of age-matched *Rgs1*$^{+/+}$ and *Rgs1*$^{-/-}$ mice**.** Frequencies (in % viable cells) of major T cell subsets isolated from distinct T cell compartments (n=13-16, analyzed in 3 independent experiments with 4-6 animals per experiment, mean +/- SEM, Mann-Whitney test, **, p <0.01.

Supplementary Figure 3

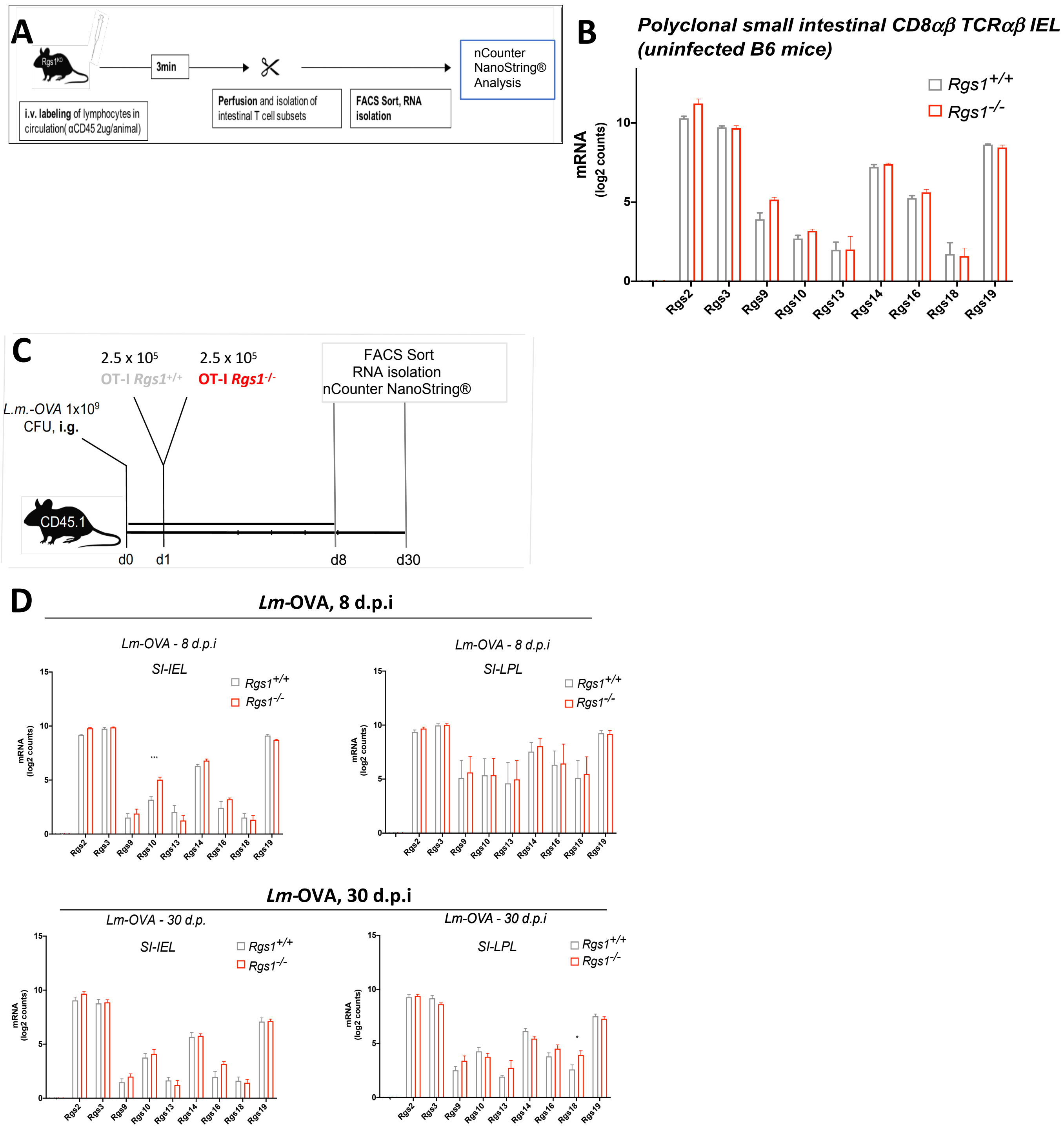

Expression of *Rgs* gene family members in T cell subsets from *Rgs1*$^{-/-}$ vs. *Rgs1*$^{+/+}$ B6 mice under non-inflammatory conditions and following intestinal infection with *Lm-OVA*

(A,B) Tissue-resident, small intestinal polyclonal CD8αβ^{+} TCRαβ IEL from *Rgs1*$^{-/-}$ vs. *Rgs1*$^{+/+}$ B6 mice were isolated and FACS sorted for subsequent mRNA analysis on a nCounter NanoString® platform for the indicated members of the Rgs-gene family (n=4 mice each, 2-way ANOVA Sidaks multiple comparisons test, all P-values > 0.05 (not significant). (C, D) Lm-OVA infected (i.g.) B6 mice were adoptively transferred with equal numbers of CD45-congenic OT-I Rgs1-/-, and OT-I Rgs1+/+ T cells. FACS-sorted OT-I Rgs1-/-, and OT-I Rgs1+/+ cells were analyzed for mRNA expression of the indicated genes on a nCounter NanoString® platform on day 8 and 30 post-infection (n=4 for 8 d.p.i and n=7 for 30 d.p.i; 2-way ANOVA Sidaks multiple comparisons test, *p <0.05, ***p <0.001, non-significant results are not shown).

Supplementary Figure 4

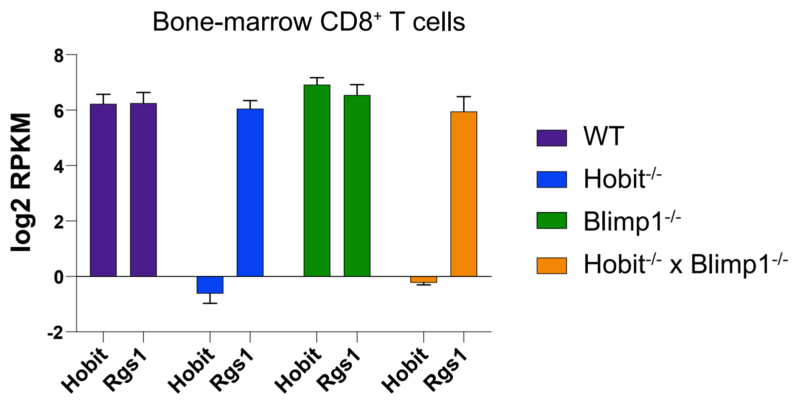

Mackay et al., Science 2016 (GSE70813)

Rgs1* expression in CD8^{+} T cells occurs independent of *Hobit/Zfp683* and *Blimp1

Rgs1 expression in *Hobit/Zfp683* and *Blimp1* deficient CD8^{+} T cells. Indicated are the log2 RPKM of *Rgs1* and *Hobit/Zfp683* mRNA in wild-type (WT), *Hobit/Zfp683* knock-out (Hobit$^{-/-}$), *Blimp1* knock-out (Blimp1$^{-/-}$) and *Hobit/Zfp683 x Blimp1* double knock-out (Hobit$^{-/-}$ x Blimp1$^{-/-}$) in primary, bone-marrow derived CD8^{+} T cells. Data were retrieved from https://www.ncbi.nlm.nih.gov, GSE70813, Mackay et al., Science, 2016.

Supplementary Figure 5

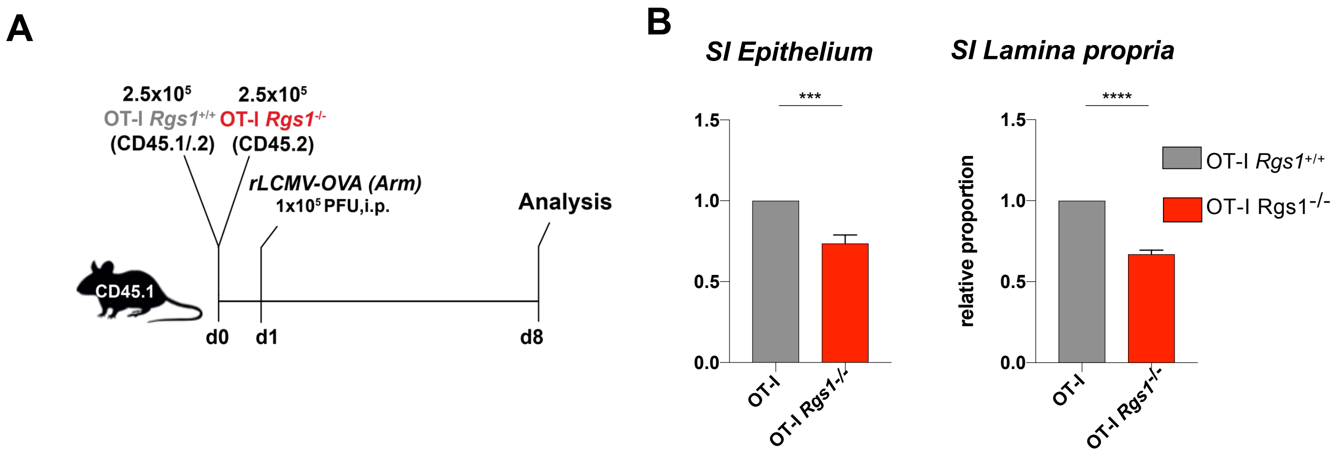

OT-I *Rgs1*$^{-/-}$ T cells become underrepresented in the small intestinal IEL and LPL compartment after systemic infection with *LCMV-OVA*

(A) Experimental set-up. A total of 5x10^{5} OT-I *Rgs1*$^{+/+}$ (CD45.1/.2) and OT-I *Rgs1*$^{-/-}$ (CD45.2) cells (1:1) were adoptively transferred i.v. into CD45.1 recipient mice. The following day recipient mice were infected i.p. with 1x10^{5} PFU *LCMV-OVA*. At day 8 p.i. OT-I *Rgs1*$^{+/+}$ and OT-I *Rgs1*$^{-/-}$ cells were isolated from the small intestine and OT-I IEL and LPL were quantitated by FACS. (**B**) Pair-wise normalized OT-I *Rgs1*$^{-/-}$ to OT-I *Rgs1*$^{+/+}$ cell ratios (OT-I Rgs1+/+ = 1.0) at day 8 after infection with LCMV-OVA (n=15, pooled from 2 independent experiments); mean ± SEM, paired Students t-test, two-tailed; ***, $p < 0.001$; ****, $p < 0.0001$.

Supplementary Figure 6

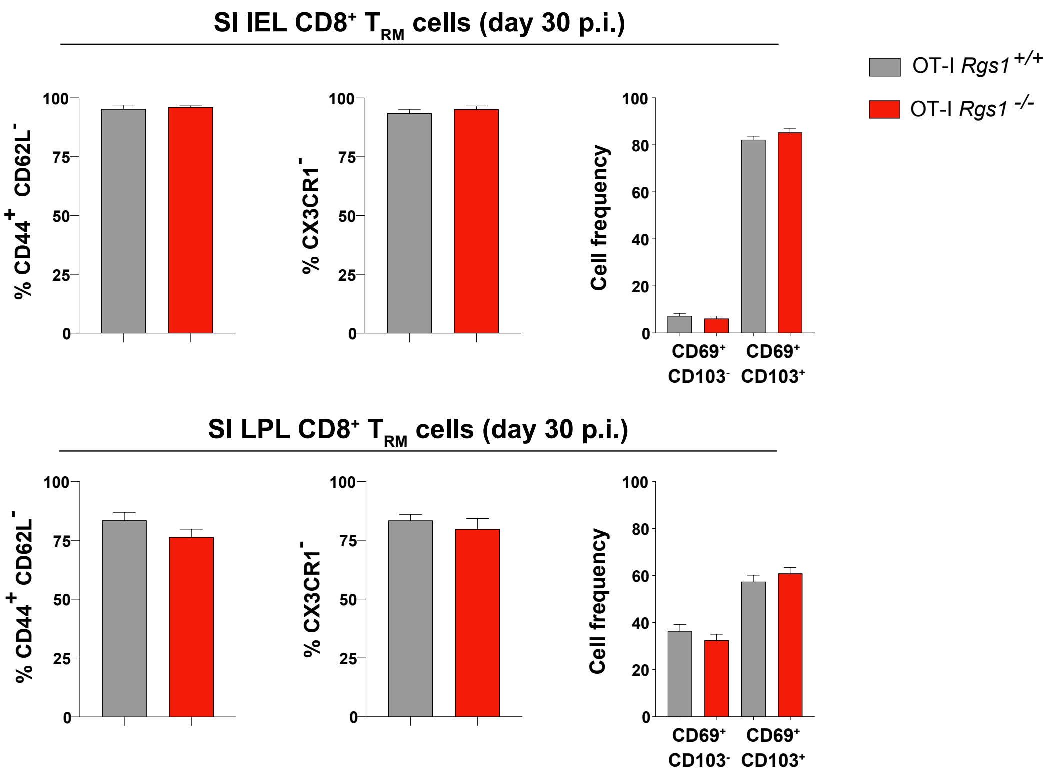

Phenotype of OT-I *Rgs1*$^{+/+}$ and OT-I *Rgs1*$^{-/-}$ CD8$^+$ T$_{RM}$ cells in the small intestine at d30 after infection with *Lm-OVA*
CD45.1 recipient mice were inoculated i.g. with $1x10^9$ CFU *Lm-OVA*. The following day a total of $5x10^5$ OT-I *Rgs1*$^{+/+}$ (CD45.1/.2) and OT-I *Rgs1*$^{-/-}$ (CD45.2) cells (1:1) were transferred (i.v.) into recipient mice. At day 30 p.i. with *Lm-OVA*, OT-I *Rgs1*$^{+/+}$ and OT-I *Rgs1*$^{-/-}$ cells were isolated and the expression of CD69, CD103, CD44, CD62L, CX$_3$CR1 was examined by flow cytometry analyzed. Frequencies of cells with the indicated surface marker profile on OT-I *Rgs1*$^{+/+}$ vs. OT-I *Rgs1*$^{-/-}$ small intestinal IEL and LPL are shown (n=17-18, pooled from 3 independent experiments, Wilcoxon test, non-significant results were not indicated).

Supplementary Figure 7

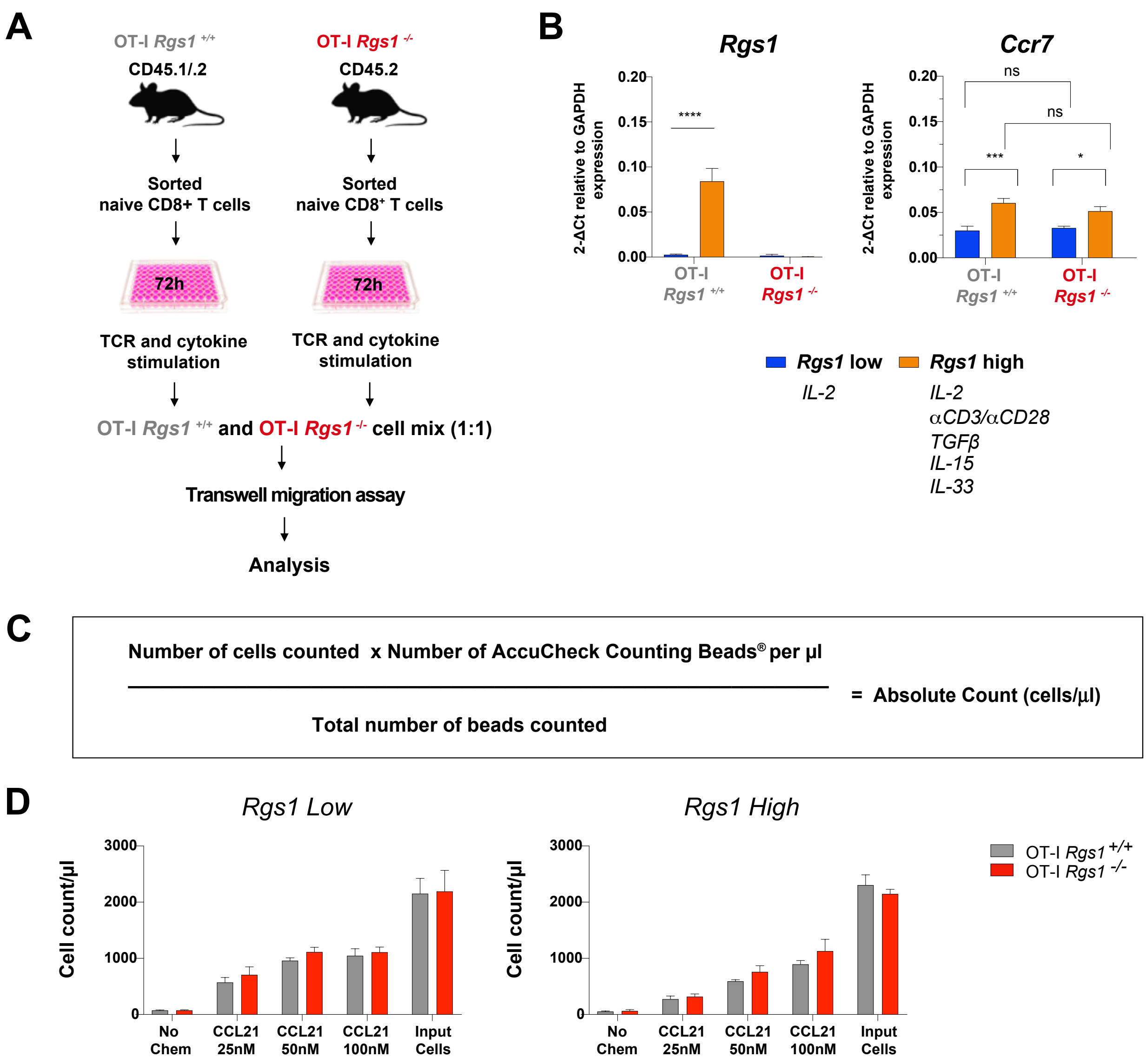

***Rgs1* does not affect the *in vitro* chemotactic migration of OT-I cells towards different concentrations of the CCR7 agonist CCL21**

(A) Experimental set-up. Naive OT-I *Rgs1*$^{+/+}$ and OT-I *Rgs1*$^{-/-}$ cells were isolated using magnetic beads and cultured in the presence of IL-2 only (non-*Rgs1* inducing conditions, "*Rgs1 Low*") or activated with anti-CD3/CD28 mAb's and IL-2, TGFβ, IL-15 and IL-33 (optimized *Rgs1*-inducing conditions "*Rgs1 High*"). After the 72h culture period, equal numbers of OT-I *Rgs1*$^{+/+}$ and OT-I *Rgs1*$^{-/-}$ cells were mixed and added into the upper chamber. The cells were allowed to migrate towards the CCL21 gradient for 2h. Migrated cells were stained for the congenic markers (CD45.1 and CD45.2) and mixed with AccuCheck Counting Beads® for quantitation by flow cytometry. **(B)** Expression of *Rgs1* and *Ccr7* in OT-I *Rgs1*$^{+/+}$ and OT-I *Rgs1*$^{-/-}$ cells, cultured under *Rgs1* inducing ("*Rgs1 High*") and *Rgs1* non-inducing ("*Rgs1 Low*") conditions (n=5, pooled from 2 independent experiments, 2-way-ANOVA with Sidak's multiple comparisons test. *, $p < 0.05$, **, $p < 0.01$, ***, $p < 0.001$, ns=not-significant). Gene expression was assesed by qPCR analysis. **(C)** OT-I Rgs1$^{+/+}$ (CD45.1/.2) and OT-I Rgs1$^{-/-}$ (CD45.2) cells and AccuCheck Counting Beads® were quantitated by flow cytometry to determine absolute numbers of gated cells according to the manufacturer's protocol. **(D)** Absolute numbers of migrated cells measured under "Rgs1 Low" and "Rgs1 High" conditions *in vitro* (n=5, pooled from 2 independent experiments, Wilcoxon test, non-significant results ($p > 0.05$) are not indicated).

Supplementary Figure 8

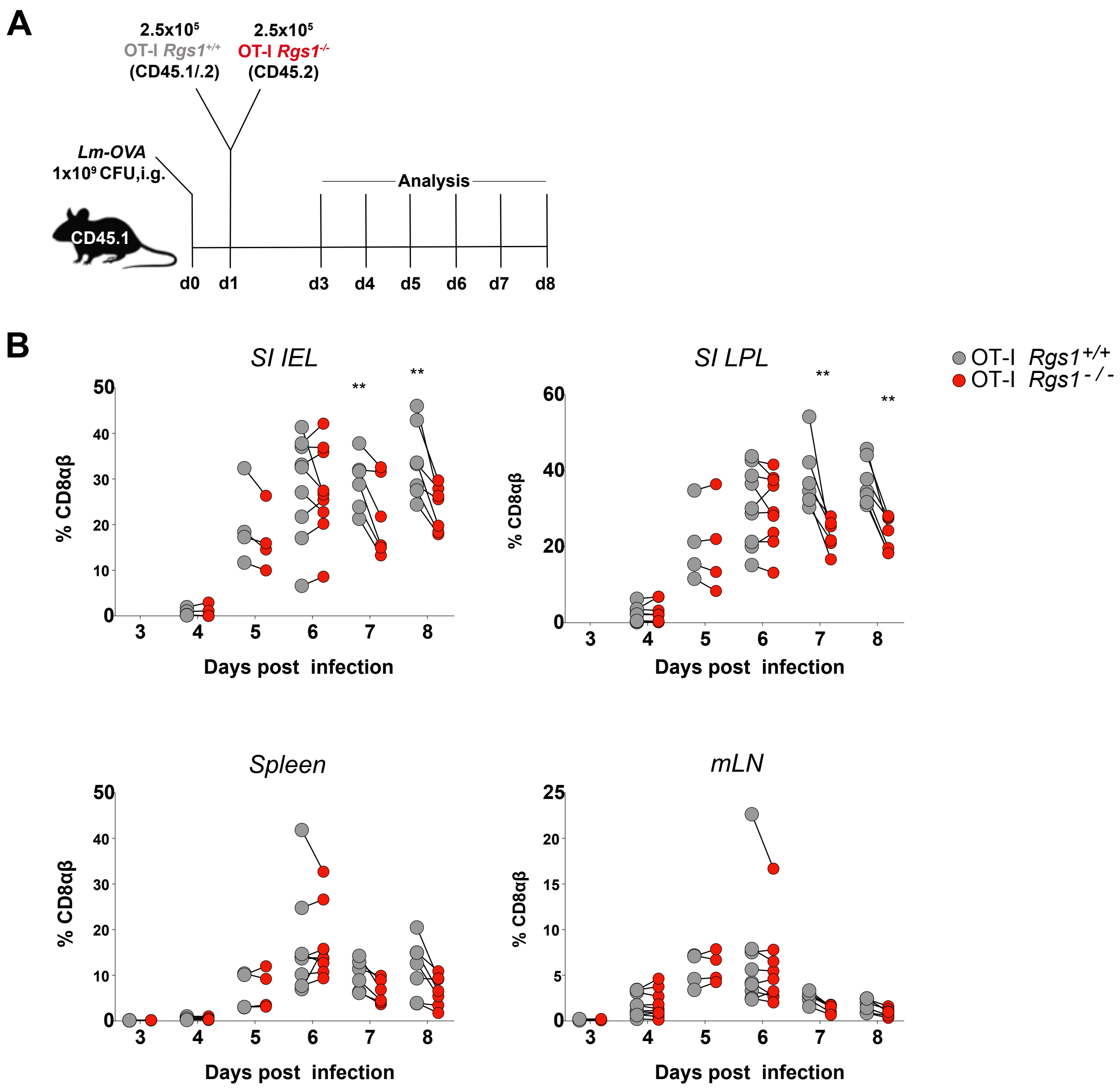

Rgs1 - deficient OT-I T cells become underrepresented after intestinal infection with *Lm*-OVA during the contraction phase

Percentage of co-transferred OT-I *Rgs1*$^{+/+}$ and OT-I *Rgs1*$^{-/-}$ cells of TCRαβ CD8αβ^{+} T cells in the small intestinal epithelium (SI IEL), lamina propria (SI LPL), spleen and mesenteric lymph node (mLN) in individual mice at the indicated days post infection with *Lm*-OVA. Values for OT-I *Rgs1*$^{+/+}$ and OT-I *Rgs1*$^{-/-}$ cells from the same individual mouse are linked (n=4 - 10 per time point, pooled data from 2 independent experiments, 2-way-ANOVA with Sidak's multiple comparisons test. **, $p < 0.01$.

Supplementary Figure 9

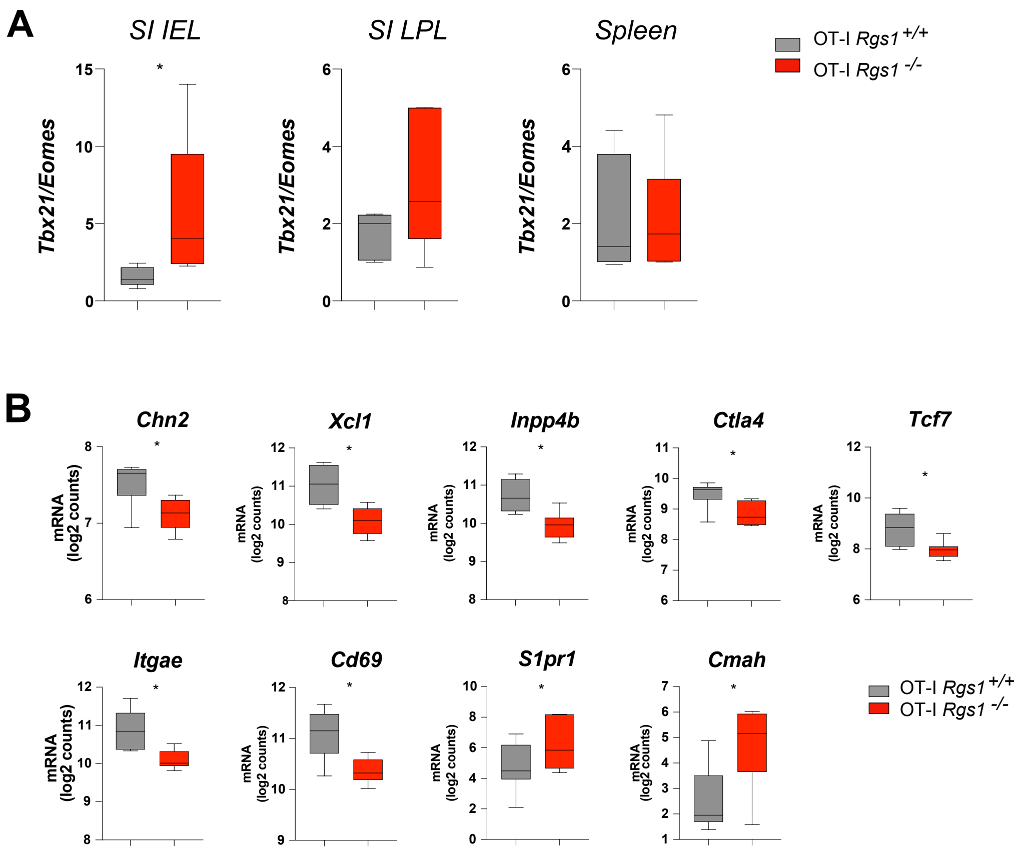

Differential expression of *Tbx21/Tbet* and *Eomes*, and distinct T_{RM} signature genes in OT-I *Rgs1*$^{+/+}$ vs. OT-I *Rgs1*$^{-/-}$ cells indicate a *Rgs1*-mediated effect on SLEC vs. MPEC in the small intestinal mucosa following infection with *Lm-OVA*

(A) *Tbx21(T-bet)/Eomes* mRNA ratio in FACS purified OT-I Rgs1$^{+/+}$ and OT-I Rgs1$^{-/-}$ CD8^{+} T cells isolated from the small intestinal lamina propria, epithelium and spleen at day 8 p.i. with *Lm-OVA* (n=5-6 pooled from 2 independent experiments) **(B)** Expression of selected CD8 T_{RM} core signature gene members known to be upregulated (*Chn2, Xcl1, Inpp4b, Ctla4,Tcf7, Itgae,* and *Cd69*) or downregulated (*S1pr1* and *Cmah*) in in T_{RM} cells are shown for OT-I *Rgs1*$^{-/-}$ vs. OT-I *Rgs1*$^{+/+}$ cells in the SI lamina propria on day 30 p.i. with *Lm OVA* (n=7-8, pooled from 2 independent experiments). For multiplex gene expression analysis, a customized NanoString® nCounter Custom CodeSet was used. Box-and-whisker plots, the box extends between 25-75% and the whiskers extend to the minimum and maximum values, Wilcoxon test *, p <0.05).

Supplementary Figure 10

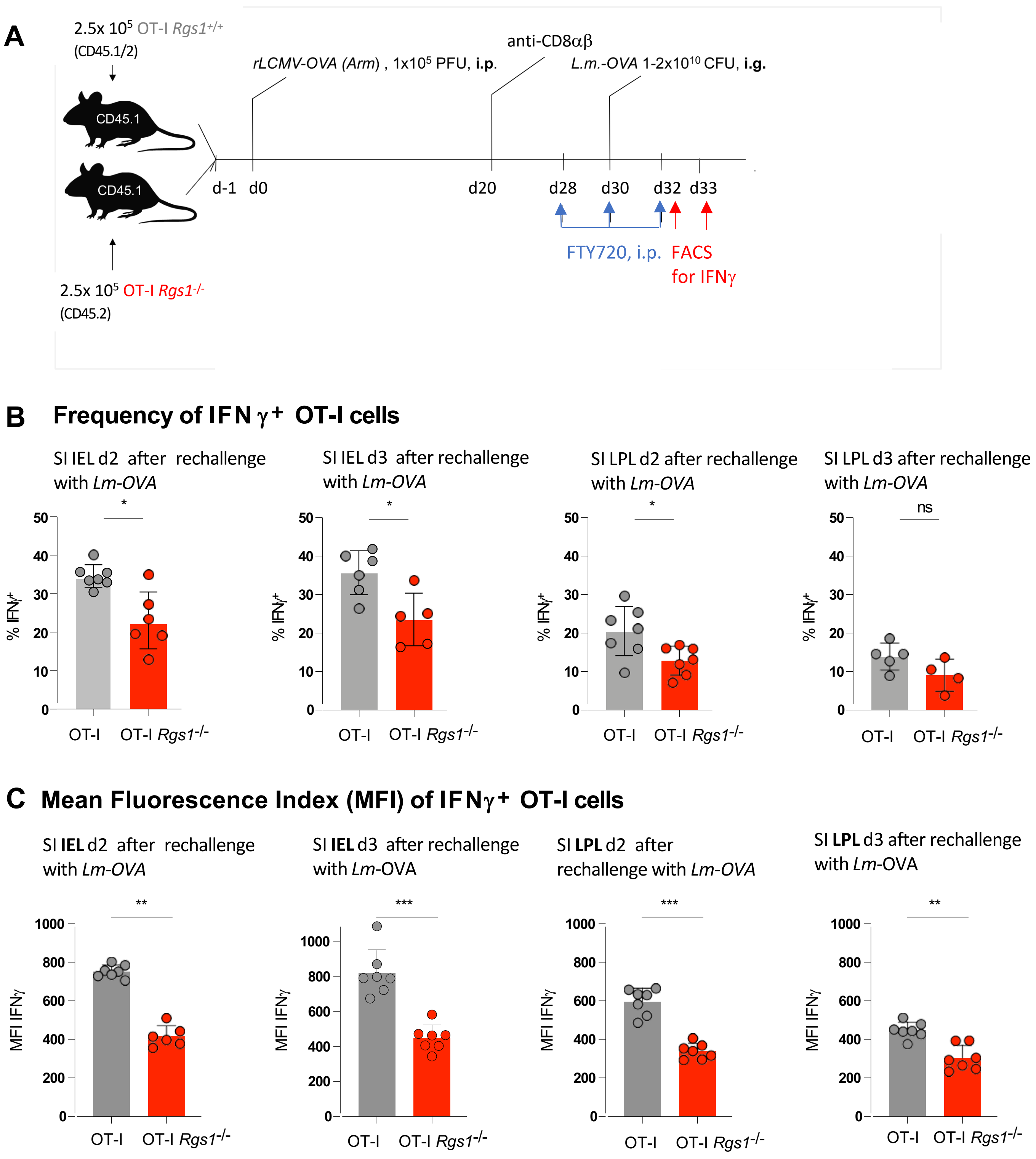

Intestinal OT-I *Rgs1*$^{+/+}$ T$_{RM}$ cells are more potent producers of IFNγ than OT-I *Rgs1*$^{-/-}$ T$_{RM}$ cells after OVA-specific rechallenge with *Lm*-OVA (i.g)

(A) Experimental set-up; OT-I cells were isolated on day 2, and day 3 after intestinal OVA-specific rechallenge of *LCMV*-OVA-primed mice with *Lm*-OVA (i.g.) to determine **(B)** the frequency of IFNγ positive small intestinal OT-I *Rgs1*$^{+/+}$ vs. OT-I *Rgs1*$^{-/-}$ cells. **(C)** Mean fluorescence index of the IFNγ^{+} OT-I cells on d2 and d3 post OVA-specific rechallenge with *Lm*-OVA; n= 4 -7; Mann-Whitney U test was used for statistical analysis; *, $p < 0.05$; **, $p < 0.01$; ***, $p < 0.001$
