# Supplementary material for: Regulator of G-protein signaling 1 critically supports CD8+ TRM cell-mediated intestinal immunity
Source: Front Immunol. 2023 Apr 20;14:1085895. doi: 10.3389/fimmu.2023.1085895 (PMC10158727; doi:10.3389/fimmu.2023.1085895)
Supplement: Supplementary file 2 [file DataSheet_2.docx]

***Von Werdt & Gungor et al. Front. Immunol. 14:1085895***

**Supplementary Material: nCounterNanostring^TM^ raw data**

The different experiments using nCounter™ Nanostring analysis can be roughly divided into the experimental groups “**Experiment #1** – **Experiment #5**”:

**Experiment #1**

**Expression of *Rgs* gene family members in small intestinal non-circulating (resident) T cell subsets** **versus circulating splenic cell subsets in C57BL/6JRj mice**

**Experimental procedure:** C57BL/6JRj mice were injected i.v. with a fluorophore (FL)-conjugated anti-CD45 mAb 3 min before euthanasia (Supplementary Figure 3A).

The following T cell subsets were used for subsequent nCounter™ Nanostring analysis using the Custom nCounter™ CodeSet (79 genes) (see: nCounter™ CodeSet Design Report for details):

- Circulating (intravital staining (iv) positive) spleen TCRαβ+ CD4+ T cells (Tab CD4 SP iv+)
- Circulating (intravital staining (iv) positive) spleen TCRαβ+ CD8αβ+ T cells (Tab CD8ab SP iv+)
- Non-circulating (intravital staining (iv) negative), tissue-resident small intestinal TCRγδ+ intraepithelial lymphocytes (**Tgd SI IEL iv-**)
- Non-circulating (intravital staining (iv) negative), tissue-resident small intestinal TCRαβ+ CD8αα+ intraepithelial lymphocytes (**Tab CD8aa SI IEL i.v.-**)
- Non-circulating (intravital staining (iv) negative), tissue-resident small intestinal TCRαβ+ CD8αβ+ intraepithelial lymphocytes (**Tab CD8ab SI IEL i.v.-**)
- Non-circulating (intravital staining (iv) negative), tissue-resident small intestinal TCRαβ+ CD4+ lamina propria lymphocytes (**Tab CD4 SI LPL i.v.-**)

**Experiment #2**

**Expression of T-cell associated genes including members of the *Rgs* gene family and of T_RM_ - signature genes in antigen-specific OT-I *Rgs1*^+/+^ T cells following infection with *L. monocytogenes*-OVA in the intestinal epithelium and lamina propria, and in the splenic central memory (T_CM_) cell compartment**

**Experimental procedure:** TCRαβ-transgenic, ovalbumin (OVA257-264) - specific OT-I CD8+T cells were transferred into CD45.1 congenic recipient mice, previously infected by an intragastric (i.g.) gavage with ovalbumin-expressing *L. monocytogenes*-OVA (1x10^9^ CFU i.g./animal). Groups of mice were sacrificed either at day 8 post-infection (d8pi), i.e. at a time when *L. monocytogenes* are largely cleared in immunocompetent mice, or during the memory phase at day 30 post-infection (30dpi) (**Figure 3A**)

The following OT-I cell subsets were isolated and FACS sorted from *L. monocytogenes-OVA* infected mice for subsequent nCounter™ Nanostring analysis using the Custom nCounter™ CodeSet (79 genes) (see: nCounter™ CodeSet Design Report for details).

***Day 8 post-infection (d8p.i) with L. monocytogenes-OVA:***

- non-circulating (intravital staining negative) OT-I cells, FACS-sorted from small intestinal intraepithelial lymphocytes (**IEL OT-I d8pi**)

- non-circulating (intravital staining negative) OT-I cells, FACS-sorted from small intestinal lamina propria lymphocytes (**LPL OT-I d8pi**)

- OT-I cells, FACS-sorted from the spleen (**SP OT-I d8pi**)

***Day 30 post-infection (30d.p.i) with L. monocytogenes-OVA:***

- non-circulating (intravital staining negative) CD69+ CD103+ OT-I cells from small intestinal intraepithelial lymphocytes (**IEL OT-I 30dpi T_RM_**)

- non-circulating (intravital staining negative) CD69+ CD103+ OT-I cells from small intestinal lamina propria lymphocytes (**LPL OT-I 30dpi T_RM_**)

- CD62L- CD44+ OT-I central memory (T_CM)_ T cells from spleen (**SP OT-I 30dpi T_CM_**)

**Experiment #3**

**Differential induction of T-cell associated genes including members of the *Rgs* gene family and of T_RM_ - signature genes in antigen-specific OT-I *Rgs1*^+/+^ vs *Rgs1*^-/-^ T cells during *in vitro* cultures of ovalbumin (OVA257-264) - specific OT-I CD8+T cells in the presence, vs. absence, of TCR/CD3 activation and distinct *Rgs1*- inducing cytokines**

**Experimental procedure:**  Naïve OT-I T cells from the spleen of naïve OT-I TCRαβ transgenic donor mice (C57BL/6JRj; *Rgs1*-/- B6) were FACS sorted and cultured for 72h in IL2 containing medium in the presence, or absence, of αCD3/αCD28, TGFβ, IL-15, and/or IL33.

The following OT-I cell cultures were used for nCounter™ Nanostring analysis using the Custom nCounter™ CodeSet (79 genes) (see: nCounter™ CodeSet Design Report for details).

- OT-I cells cultured for 72h *in vitro* under non-Rgs1 inducing conditions, i.e. in the presence of IL2 only (**Rgs1lo WT OT-I IL-2**)
- OT-I cells cultured for 72h *in vitro* under Rgs1 inducing conditions, i.e. either in the presence of IL2, anti-CD3/CD28 mAb, TGFβ, and IL33 (**Rgs1hi WT OT-I TCR, TGFb IL33**) or in the presence of IL2, anti-CD3/CD28 mAb, TGFβ, and IL15 (**Rgs1hi WT OT-I TGFb IL15**)

**Experiment #4**

**Expression of T-cell associated genes including members of the *Rgs* gene family members and of T_RM_ - signature genes in polyclonal small intestinal CD8αβ+ TCRαβ IEL from *Rgs1*^-/-^ vs. *Rgs1*^+/+^ B6 mice under non-inflammatory conditions.**

**Experimental procedure:**  C57BL/6JRj donor mice (WT) and *Rgs1*-/- B6 mice (Rgs1 -/- B6) were injected i.v. with a fluorophore (FL)-conjugated anti-CD45 mAb 3 min before euthanasia (Supplementary Figure 3A). Non-circulating, i.e. intravital staining-negative (i.v.-) polyclonal small intestinal CD8αβ+ TCRαβ + intraepithelial T cells from Rgs1+/+ donor mice (**Tab CD8ab SI IEL i.v.- (WT)** and from Rgs1-/- mice (**Tab CD8ab SI IEL i.v.- (Rgs1 -/- B6)** were FACS-sorted for subsequent nCounter™ Nanostring analysis using the Custom nCounter™ CodeSet (see: nCounter™ CodeSet Design Report for details).

**Experiment #5**

**Expression of T-cell associated genes including members of the *Rgs* gene family and of T_RM_ - signature genes in antigen-specific OT-I *Rgs1*+/+ vs *Rgs1*-/- T cells following infection with *L. monocytogenes*-OVA**

**Experimental Procedure:** *L. monocytogenes*-OVA infected (i.g.) B6 mice adoptively received equal numbers of CD45-congenic OT-I *Rgs1*-/-, and OT-I *Rgs1*+/+ T cells (Figure 4A). On day 8, and day 30 post-infection, mice were first injected i.v. with a fluorophore (FL)-conjugated anti-CD45 mAb 3 min before euthanasia (Supplementary Figure 3A) to distinguish circulating (stained) from non-circulating cells (unstained).

Non-circulating, i.e. intravital staining-negative (i.v.-) FACS-sorted OT-I *Rgs1*-/-, and OT-*I Rgs1*+/+ cells were used for subsequent nCounter™ Nanostring analysis

In one sub-experiment (RCC file 20180221 and 20180227) non-circulating, i.e. intravital staining-negative (i.v.-) OT-I *Rgs1*-/-, and OT-*I Rgs1*+/+ cells, which also stained positive for CD103 were FACS sorted for subsequent nCounter™ Nanostring analysis using the Custom nCounter™ CodeSet.

Collectively, the following sorted OT-I cells were included for nCounter™ Nanostring analysis using the Custom nCounter™ CodeSet (see: nCounter™ CodeSet Design Report for details).

***Day 8 post-infection (d8p.i) with L. monocytogenes-OVA:***

- non-circulating (intravital staining negative) OT-I *Rgs1*+/+ cells isolated and FACS-sorted from small intestinal intraepithelial lymphocytes on day 8 post-infection with *L. mono-OVA* (**IEL OT-I d8pi**)

- non-circulating (intravital staining negative) OT-I *Rgs1*-/- cells isolated and FACS-sorted from small intestinal intraepithelial lymphocytes on day 8 post-infection with *L. mono-OVA* (**IEL OT-IxRgs1Ko d8pi**)

- non-circulating (intravital staining negative) OT-I *Rgs1*+/+ cells isolated and FACS-sorted from small intestinal lamina propria lymphocytes on day 8 post-infection with *L. mono-OVA* (**LPL OT-I d8pi**)

- non-circulating (intravital staining negative) OT-I *Rgs1*-/- cells isolated and FACS-sorted from small intestinal lamina propria lymphocytes on day 8 post-infection with *L. mono-OVA* (**LPL OT-IxRgs1Ko d8pi**)

- OT-I *Rgs1*+/+ cells isolated and FACS-sorted from the spleen on day 8 post-infection with *L. mono-OVA* **(SP OT-I d8pi)**

- OT-I *Rgs1*-/- cells isolated and FACS-sorted from the spleen on day 8 post-infection with *L. mono-OVA* (**SP OT-IxRgs1Ko d8pi**)

***Day 30 post-infection (30d.p.i) with L. monocytogenes-OVA:***

- non-circulating (intravital staining negative) OT-I *Rgs1*+/+ cells isolated and FACS-sorted from small intestinal intraepithelial lymphocytes on day 30 post-infection with *L. mono-OVA* (**IEL OT-I d30pi TRM**)

- non-circulating (intravital staining negative) OT-I *Rgs1*-/- cells isolated and FACS-sorted from small intestinal intraepithelial lymphocytes on day 30 post-infection with *L. mono-OVA* (**IEL OT-IxRgs1Ko d30pi TRM**)

- non-circulating (intravital staining negative) OT-I *Rgs1*+/+ cells isolated and FACS-sorted from small intestinal lamina propria lymphocytes on day 30 post-infection with *L. mono-OVA* (**LPL OT-I d30pi TRM**)

- non-circulating (intravital staining negative) OT-I *Rgs1*-/- cells isolated and FACS-sorted from small intestinal lamina propria lymphocytes on day 30 post-infection with *L. mono-OVA* (**LPL OT-IxRgs1Ko d30pi TRM)**

- non-circulating, i.e. intravital staining-negative (i.v.-) OT-*I Rgs1*+/+ cells, which also stained positive for CD103 were isolated from the small intestinal lamina propria on day 30 post-infection with *L. mono-OVA* (**LPL OT-I CD103+ 30d p**.i.)

- non-circulating, i.e. intravital staining-negative (i.v.-) OT-I *Rgs1*-/- cells, which also stained positive for CD103 were isolated from the small intestinal lamina propria on day 30 post-infection with *L. mono-OVA* (**LPL OT-IxRgs1Ko CD103+ 30d p.i.)**
